# Genetic Factors Associated With Tardive Dyskinesia: From Pre-clinical Models to Clinical Studies

**DOI:** 10.3389/fphar.2021.834129

**Published:** 2022-01-24

**Authors:** Evangelia Eirini Tsermpini, Sara Redenšek, Vita Dolžan

**Affiliations:** Pharmacogenetics Laboratory, Institute of Biochemistry and Molecular Genetics, Faculty of Medicine, University of Ljubljana, Ljubljana, Slovenia

**Keywords:** tardive dyskinesia, antipsychotics, animal models, pharmacogenetics, GWAS

## Abstract

Tardive dyskinesia is a severe motor adverse event of antipsychotic medication, characterized by involuntary athetoid movements of the trunk, limbs, and/or orofacial areas. It affects two to ten patients under long-term administration of antipsychotics that do not subside for years even after the drug is stopped. Dopamine, serotonin, cannabinoid receptors, oxidative stress, plasticity factors, signaling cascades, as well as CYP isoenzymes and transporters have been associated with tardive dyskinesia (TD) occurrence in terms of genetic variability and metabolic capacity. Besides the factors related to the drug and the dose and patients’ clinical characteristics, a very crucial variable of TD development is individual susceptibility and genetic predisposition. This review summarizes the studies in experimental animal models and clinical studies focusing on the impact of genetic variations on TD occurrence. We identified eight genes emerging from preclinical findings that also reached statistical significance in at least one clinical study. The results of clinical studies are often conflicting and non-conclusive enough to support implementation in clinical practice.

## 1 Introduction

Psychiatric disorders affect approximately 30% of the population worldwide (James et al., 2018). They can be treated with a combination of different drugs, such as antipsychotics, antidepressants, mood stabilizers, and anxiolytic drugs. According to the National Health and Nutrition Examination Survey (NHANES) data collected between 2013 and 2018, which included 17,691 U.S. residents, the estimated prevalence of antipsychotic drug use was 1.6% (Dennis et al., 2020). In 2020, antipsychotic drugs were prescribed more than 5 million times (Dennis et al., 2020). The treatment of psychiatric disorders is a challenge, and the drug efficacy is limited. Moreover, the administration of these drugs is usually accompanied by the occurrence of mild to serious adverse events (Reynolds 2007). Despite large number of typical and atypical antipsychotic drugs, it is difficult to find the safest drug with the maximum therapeutic efficacy for the patient. Additionally, patients treated with antipsychotics were at higher risk for comorbidities (Dennis et al., 2020). This can lead to polypharmacy and high risk of unpleasant adverse events (Ravyn et al., 2013).

Tardive dyskinesia (TD) is a severe motor adverse event of antipsychotic medication, characterized by involuntary athetoid movements of the trunk, limbs, and/or orofacial areas that affects 25.3% of patients under long-term treatment with antipsychotics. (Owens 2019; Islam et al., 2021). According to the Diagnostic and Statistical Manual of Mental Disorders, Fifth Edition (DSM-V), tardive dyskinesia is a drug-induced movement disorder that lasts for 1 month (American Psychiatric Association 2013). It is also a very persistent disorder, meaning that its symptoms do not subside for years even after the drug is stopped (Vasan and Padhy 2021) It decreases patients’ quality of life and leads to difficulties in daily life and stigmatization (Owens 2019). TD severity is assessed with the Abnormal Involuntary Movement Scale (AIMS). This 12-item scale can serve as an essential tool for TD diagnosis in clinical practice when used by trained physicians and healthcare professionals. The first items refer to movements of different body regions, such as facial and oral areas, extremities, and trunk. The rest reflect the global judgment regarding movement severity and accounts for dental status (Guy 1976; Caroff et al., 2020). According to Schooler and Kane criteria, the prerequisites for TD diagnosis include at least 3 months of exposure to an antipsychotic drug, moderate abnormal movements in one body part or mild in two or more, and no other potential cause (Schooler and Kane 1982).

The development of TD is related to demographic variables, such as age, sex, and race, as well as the class of antipsychotics prescribed, the duration, and the dosage of the antipsychotic treatment (Zai et al., 2018b). Women, elderly patients, African Americans, patients with mood disorders, intellectual disability, or central nervous system injury, as well as patients with a past or current history of akathisia, parkinsonism, or acute dystonic reactions, are more susceptible to developing TD (Owens 2019; Keepers et al., 2020; Vasan and Padhy 2021). TD is caused mainly by the long-term exposure to antipsychotics, either first-generation (FGAs) or second-generation (SGAs) (Lee and Kang 2011; Zai et al., 2018b; Owens 2019). However, 20% of patients treated with FGAs experience TD. One of the underlying causes is the increased dopamine D2 affinity that characterizes FGAs (Vasan and Padhy 2021). On the contrary, the lower D2 affinity of SGAs, such as clozapine or quetiapine, might improve TD symptoms (Ricciardi et al., 2019; Vasan and Padhy 2021). Notably, a 12-years cross-national study that inspected the patterns of antipsychotic prescription in Taiwan, Hong Kong, Japan, and the United States, indicated that SGAs were more frequently prescribed in younger patients in all studied countries. Quetiapine and haloperidol were the most common in the United States and Hong Kong (Su et al., 2020). It is crucial to mention that among patients receiving an antipsychotic, only 1 out of 10 had a schizophrenia spectrum disorder diagnosis, while the rest had bipolar disorder, treatment-resistant depression, anxiety, personality disorders, autism, and/or other conditions (Loughlin et al., 2019; Dennis et al., 2020).

Although the results of two randomized controlled trials (RCTs) indicated that there was no association between the class of antipsychotics and TD frequency (Miller et al., 2008; Peluso et al., 2012), a meta-analysis published in 2018, which assessed 57 RCTs, suggested that the administration of SGAs is associated with lower risk for TD development (Carbon et al., 2018). Switching from FGAs to SGAs was proposed as a more efficient strategy for improvement of symptoms than treatment discontinuation (Bai et al., 2003) and a later systematic review confirmed a reduction of TD symptoms (Ricciardi et al., 2019). However, the 18-years long prospective study indicated that adding a SGA to existing FGA treatment was not necessarily associated with reduction in TD severity (Mentzel et al., 2017). The results are conflicting regarding the impact of duration and the dosage of the antipsychotic therapy on TD risk. A retrospective study suggested that the dose of antipsychotics was associated with high TD risk in patients with schizophrenia (Patterson-Lomba et al., 2019). In contrast, a meta-analysis of 26 studies (Takeuchi et al., 2020) and a review of 13 RCTs (Bergman et al., 2018) did not confirm this association.

The Curaçao Extrapyramidal Syndromes Study XII, a prospective study that lasted for 18 years and included 223 patients, suggested that an increase in the dose of antipsychotics can reduce the severity of TD (Mentzel et al., 2017). This finding is in line with other older studies with a small sample size (Kazamatsuri et al., 1972, Kazamatsuri et al., 1973; Gerlach et al., 1974; Korsgaard et al., 1984; Tamminga et al., 1994). However, attention should be paid to the potential masking effect of the increased dose on TD symptoms, and it should be emphasized that the alleviation is only short-term and that TD symptoms inevitably return after some time (Mentzel et al., 2017; Yoshida and Takeuchi 2021).

Age, sex, health behaviors, liver and renal function, comorbidities, and the administration of co-medication are important factors in choosing the right type of the antipsychotic drug and its dose (Ohmori et al., 2003; Ravyn et al., 2013). Apart from the above factors a very crucial variable of TD development is individual susceptibility and genetic predisposition to TD development. Dopamine, serotonin, cannabinoid receptors, oxidative stress, plasticity factors, signaling cascades, as well as CYP isoenzymes and transporters have been associated with TD occurrence, in terms of genetic variability, and metabolic capacity. The genetic variability of pharmacokinetic and pharmacodynamic genes can lead to decreased or increased drug plasma levels and impact the drug response, in terms of efficacy and toxicity (Ravyn et al., 2013).

To elucidate potential genetic factors contributing to the molecular pathogenesis of TD, this review first collected all the available data on preclinical animal studies of TD development induced by antipsychotic treatment published in the last 10 years. To achieve our aim, we performed a literature review of studies published until today found in the MEDLINE database, using combinations of the following keywords: “pharmacogenomics,” “pharmacogenetics,” “tardive dyskinesia,” “drug-induced tardive dyskinesia,” “antipsychotics,” “adverse drug reactions,” “genes,” “genetic variations,” “polymorphisms,” and limited the search filter “species” to “humans.” The respective search for preclinical animal studies was performed by using the search term “tardive dyskinesia” and limiting the search filter “species” to “other animals.” Additional references were also identified and retrieved from the articles that emerged from the search using the above combinations. Next, we reviewed candidate gene and genome-wide association studies that focused on genetic variations in genes involved in pharmacokinetics and pharmacodynamics and drug-induced TD. The findings of larger, genome-wide association studies (GWAS) were also included. We elaborated on how this information can be used for patients’ benefit.

## 2 Preclinical Studies of Tardive Dyskinesia Development

Candidate genes that would be relevant to be studied in regards to TD development could be derived from preclinical studies conducted primarily on experimental animals, such as mice, rats, and monkeys (Blanchet et al., 2012). In most models, TD is induced by treatment with the FGA haloperidol, commonly prescribed in patients with schizophrenia (Marder and Cannon 2019). Additionally, fluphenazine, one of the oldest antipsychotics (Matar et al., 2018) and reserpine, an indole alkaloid used as antipsychotic and antihypertensive agent (Soung et al., 2018) are used to induce TD in experimental animals as well. The phenotype of TD in animals is different in comparison to human beings since it presents with robust, seemingly purposeless chewing activity (so-called vacuous chewing movements or VCM), often fluctuating, and occurring in short bursts, occasionally associated with bruxism and tongue protrusions. VCM in animals usually occur shortly after treatment initiation or even after acute treatments, whereas in humans the development of TD takes longer (Blanchet et al., 2012). Many studies have been conducted in experimental animals to elucidate the underlying pathology of TD and determine potential pharmacotherapy. However, no consensus has been made so far.

We identified 43 animal studies dealing with TD pathogenesis and possible ways of treatment in the past 10 years. All of the studies are presented in the Supplementary Table S1. Animal experiments studied candidate pathways involved in TD development on the level of functional perturbations due to treatment with antipsychotics. Inflammation (Bishnoi and Boparai 2012; Grover et al., 2013; Thakur et al., 2015; Datta et al., 2016; Peroza et al., 2016; Sonego et al., 2018; Soung et al., 2018; Wang et al., 2021a) and oxidative stress defense (Patil et al., 2012; Grover et al., 2013; Nade et al., 2013; Thakur et al., 2015; Wang et al., 2015; Datta et al., 2016; Schaffer et al., 2016; Samad and Haleem 2017; Dhingra et al., 2018; Sonego et al., 2018; Soung et al., 2018; Tsai et al., 2019; Wang et al., 2021a) pathways were among the most studied ones. Accordingly, different antioxidants were tested as potential agents for the treatment of TD. Many studies showed decreased levels of catalase (CAT), superoxide dismutase (SOD), and glutathione (GSH), either in their quantity or antioxidant capacity, upon treatment with haloperidol (Grover et al., 2013; Thakur et al., 2015; Datta et al., 2016; Samad and Haleem 2017; Dhingra et al., 2018; Sonego et al., 2018; Tsai et al., 2019; Wang et al., 2021a), reserpine (Patil et al., 2012; Nade et al., 2013; Wang et al., 2015; Soung et al., 2018) or fluphenazine (Schaffer et al., 2016). Studies have also shown that a plethora of cytokines are elevated upon treatment with antipsychotics, such as TNF-*α* (Bishnoi and Boparai 2012; Grover et al., 2013; Thakur et al., 2015; Datta et al., 2016; Peroza et al., 2016; Sonego et al., 2018; Soung et al., 2018; Wang et al., 2021a), IL-1*β* (Grover et al., 2013; Thakur et al., 2015; Datta et al., 2016; Peroza et al., 2016; Sonego et al., 2018; Wang et al., 2021a), IL6 (Datta et al., 2016; Peroza et al., 2016; Sonego et al., 2018; Soung et al., 2018; Wang et al., 2021a), and IFN-*γ* (Peroza et al., 2016). On the contrary, IL-10 is decreased, due to its role as an anti-inflammatory cytokine (Peroza et al., 2016; Sonego et al., 2018). Consequently, some agents with antioxidative and anti-inflammatory properties were tested as potential drugs for TD, such as resveratrol (Busanello et al., 2012, Busanello et al., 2017), vitamin E (An et al., 2016; Shi et al., 2016), lycopene (Datta et al., 2016), L-theanine (Chen et al., 2018; Soung et al., 2018; Tsai et al., 2019), cannabidiol (Sonego et al., 2018; Kajero et al., 2020), and catechin (Wang et al., 2015; Reinheimer et al., 2020). Additionally, several different plant-based compounds were tested (Patil et al., 2012; Sekiguchi et al., 2012; An et al., 2013; An et al., 2016; Shi et al., 2016; Samad and Haleem 2017; Dhingra et al., 2018; Wang et al., 2021a). A widely measured endpoint upon TD induction is the severity of apoptosis in different brain areas. Caspase-3 was elevated in animals with TD (Bishnoi and Boparai 2012; Soung et al., 2018; Wang et al., 2021a). Furthermore, the transcription level of the proapoptotic *BAX* was elevated, whereas antiapoptotic *BAD* mRNA was decreased (An et al., 2016). In light of this brain-derived neurotrophic factor (BDNF), a molecule involved in the viability of neurons, was shown to be decreased in several brain regions of rats with haloperidol-induced VCM. It was rescued with antioxidant-acting Ginkgo biloba extract and vitamin E (Shi et al., 2016).

Since TD arises from perturbations in neurotransmitter systems, a lot of emphasis was put on that in terms of end-point measurements when TD was induced in experimental animals. Several different neurotransmitter systems were studied, such as dopaminergic, serotonergic, cholinergic, and glutamatergic. As expected, the most widely studied pathway is the dopaminergic pathway. The binding capacities or expression levels of different components of dopaminergic pathways were assessed upon haloperidol treatment. It was shown that the function or activity of dopamine transporter (Bordia et al., 2012; Lévesque et al., 2017), monoamine oxidase B (MAO-B) (Busanello et al., 2017), and dopamine receptor 1 (DRD1) (Mahmoudi et al., 2014) were decreased, whereas binding capacities or activities of the tyrosine hydroxylase (TH) (Lévesque et al., 2017; Ceretta et al., 2018) and dopamine receptor 3 (DRD3) (Mahmoudi et al., 2014; Hernandez et al., 2019) were increased. The storage capacity of the monoamine neurotransmitters was decreased since the expression of vesicular monoamine transporter 2 (VMAT2) was shown to be reduced upon TD induction (Lévesque et al., 2017). The serotonergic system works hand in hand with the dopaminergic system also in the context of TD development (Crisafulli et al., 2013) (34). The serotonin receptor 2A (HTR2A) mRNA was decreased upon chronic exposure to haloperidol in monkeys (Lévesque et al., 2017). All of the above indicate a lack of adaptation of the aminergic neurotransmission in TD development (Lévesque et al., 2017; Hauser and Truong 2018). Not only were those components of serotonergic and dopaminergic systems dysregulated, but the concentrations of neurotransmitters themselves were decreased (Dhingra et al., 2018; Soung et al., 2018; Wang et al., 2021a).

Studies of TD in animal models showed that other neurotransmitter systems, such as acetylcholine and glutamate, are also affected. Neuronal acetylcholine receptor subunits, for instance alpha-4 (CHRNA4), beta-2 (CHRNB2), and alpha-6 (CHRNA6), were decreased upon haloperidol treatment (Bordia et al., 2012). Additionally, the acetylcholinesterase activity was decreased in different brain areas in haloperidol-induced orofacial dyskinesia in rats, recuperated by the vitamin B cocktail (de Oliveira et al., 2013). On the other hand, glutamate receptor subunits, GRIN2A and GRIN2B were increased in the putamen of haloperidol treated monkeys (Lévesque et al., 2017). Along with that, glutamate transporter (SLC1A2) was decreased in the striatum and cortex of haloperidol-treated rats (Sekiguchi et al., 2012). Furthermore, cannabinoid receptor type 1 (CNR1) was shown to be increased in haloperidol-induced orofacial dyskinesia in rats (Röpke et al., 2021).

A few animal studies explored the genetic background of TD as well. Crowley et al. conducted a genome-wide association study in 27 inbred strains of mice treated with haloperidol for 60 days. TD was recorded in case the animal developed VCM. The association mapping analysis highlighted six genes with variants associated with the development of VCM, namely *CPEB2, BST1, PIT2, ZIC4, PLSCR1*, and *DRD1A*, of which the latter was concluded to be the most important one (Crowley et al., 2012).

TD was also induced in knock-out animals to dissect the role of individual genes in the development of TD. Khan et al. induced TD in the *GNAL* knock-out and wild-type mice with haloperidol. *GNAL* encodes G*α*(olf), the *α* subunit of a heterotrimeric GTP-binding protein that couples to downstream signaling partners. G*α*(olf) is highly enriched in the striatum, where it positively couples with DRD1 and ADORA2A to activate adenylyl cyclase, thereby increasing intracellular cAMP levels in DRD1-expressing striatonigral and DRD2-expressing striatopallidal neurons, respectively. G*α*(olf) levels serve as a determinant of cAMP signal-dependent activity. *GNAL* ± mice presented higher levels of DNA damage and cell death in the brain. This was accompanied by reduced levels of cAMP and histone H3 phosphorylation in the striatum and enhanced behavioral abnormalities after haloperidol treatment in comparison to *GNAL* ^+/+^ haloperidol treated mice (Khan et al., 2019). Results of this study warrant further functional research on the role of G*α*(olf) in TD.

Animal studies focusing on a particular protein function in TD development have been published as well. A study conducted by Nagaoka et al. studied the role of striatal transient receptor potential vanilloid 1 (TRPV1) in acetaminophen treatment of orofacial dyskinesia induced by haloperidol in rats. First, they successfully showed that acetaminophen and the acetaminophen metabolite AM404 are valid options for alleviation of TD. They also showed that TRPV1 is a crucial component in the mechanism of acetaminophen action since the anti-dyskinetic effect was lost in the TRPV1 knock-out animals if compared to wild-type rats (Nagaoka et al., 2021). Furthermore, in a monkey model of TD induced by haloperidol, monkeys treated with haloperidol without TD had a higher expression of the transcription factor NUR77. This indicates that NUR77, which is involved in a neuroadaptive response mounted against abnormal motor behaviors following typical antipsychotic drug exposure, is associated with a decreased risk for TD development (Mahmoudi et al., 2013). S100B, a calcium-binding protein, exerts neuroprotective and neurodegenerative effects depending on its concentration. Low concentrations appear to have neuroprotective effects on neuronal growth and survival. On the other hand, higher concentrations induce neurodegeneration and apoptosis. A rat model of TD showed increased levels of S100B, which were rescued by antioxidant administration (An et al., 2016). A study conducted by Miksys et al. dealt with brain metabolism levels of haloperidol and their association with TD development in a rat model. They show that lower CYP2D6 activity in the brain decreases the risk for catalepsy and VCM development, whereas high CYP2D6 activity increases VCM and catalepsy development (Miksys et al., 2017).

Preclinical studies showed many insights into the pathogenesis of TD and, more importantly, many suggested how this troublesome adverse event of antipsychotic treatment could be tackled. However, there are some caveats to the animal models of TD. The phenotype of TD in animals differs from the one in human beings, since TD in animals presents mostly as VCM focused on the orofacial area whereas in humans it affects other body parts as well. Additionally, VCM are often observed after >2–3 weeks of subchronic haloperidol treatment, which is a relatively long time in the average life span of a rat (Kulkarni and Dhir 2011). The percentage of animals developing VCM due to antipsychotic treatment is much higher than the percentage in patients as well (Blanchet et al., 2012). All of the above indicates that animal studies cannot substitute the findings from studies in human beings. However, they present an invaluable basis for the direction of human studies towards a better understanding and management of TD.

## 3 Pharmacogenomic Studies Focusing on the Association Between Tardive Dyskinesia and Genes Involved in the Pharmacokinetics of Antipsychotics

Pharmacogenetics aims to provide the appropriate drug for the individual patient in terms of efficacy and adverse events’ occurrence based on the patient’s genetic background (Ravyn et al., 2013). For many years the main focus of the research was genetic variability of drug metabolizing enzymes, mainly cytochromes P450 (Ohmori et al., 2003; Gopisankar 2017). The metabolizing properties and capacities of cytochromes vary a lot and they can metabolize diverse substrates (Ravyn et al., 2013; Gopisankar 2017).

The most important as well as highly polymorphic drug-metabolizing enzyme is CYP2D6. Interestingly, 30% of the drugs are primarily or partially metabolized by CYP2D6. Psychiatric drugs, including antipsychotics are among them (Petrović et al., 2020). Depending on the genotype, the metabolizing phenotype differs, and the drug metabolism consequently changes. More specifically, based on their metabolizing capacity, individuals can be grouped into four different drug metabolizer phenotypes: ultra-rapid metabolizers (UM), normal (extensive) metabolizers (EM), intermediate metabolizers (IM), and poor metabolizers (PM) (Ravyn et al., 2013; Gopisankar 2017). As the frequency of *CYP2D6* alleles differs among races and populations, the drug metabolism phenotypes distributions are different among ethnicities (Gopisankar 2017).

Researchers have extensively explored the association between *CYP2D6* genetic variability and TD development; however, the results are contradictory (Table 1 and Supplementary Table S2). Some studies did not show an association between TD and CYP2D6*1, CYP2D6*2, CYP2D6*3, CYP2D6*4, CYP2D6*5, CYP2D6*6, CYP2D6*15a, and CYP2D6*17 (Arthur et al., 1995; Armstrong et al., 1997; Ohmori et al., 1998; Ohmori et al., 1999; Brockmöller et al., 2002; Lohmann et al., 2003), but some studies indicated a statistically significant or borderline association between *CYP2D6* alleles and TD. A statistically significant association between CYP2D6*10 and TD, both before and after adjustment for clinical variables, was reported in a cohort of 100 Japanese patients with schizophrenia (Ohmori et al., 1998). Similarly, the T allele of the CYP2D6*10 was associated with TD development in a different study (Fu et al., 2006). Another study reported potential associations between CYP2D6*3, CYP2D6*4, and CYP2D6*5 variants and TD occurrence. According to the results, heterozygotes in particular presented with a higher risk for TD development (Kapitany et al., 1998). Similarly, heterozygotes for CYP2D6*3 or CYP2D6*4 had a higher chance for TD development (Jaanson et al., 2002). A report by Andreassen et al. (1997) investigated CYP2D6*1, CYP2D6*3, CYP2D6*4, CYP2D6*5, CYP2D6*6 and CYP2D6*7 variants in 100 schizophrenic patients from South-East Scotland. They showed a positive correlation between PM and TD severity (Andreassen et al., 1997). Koola et al., 2014 explored the impact of CYP2D6*3, CYP2D6*4, CYP2D6*5 and CYP2D6*41 genetic variability on TD occurrence and revealed that an increase in the number of functional CYP2D6 alleles is associated with TD risk (Koola et al., 2014). One of the latest studies with a slightly higher number of participants showed that CYP2D6*4 was associated with a higher risk for limbo-truncal TD (Ivanova et al., 2016a). Additionally, a study done by Lu et al. reported that *CYP2D6* UM and PM have a higher risk for TD development and higher severity of TD compared to IMs and EMs. This is the first study that suggested that both extremes of CYP2D6 metabolic capacity have increased TD risk instead of PM only (Lu et al., 2020). Lastly, a meta-analysis that included 12 pharmacogenetic studies aimed to identify the potential association between *CYP2D6* alleles and TD development. A statistically significant association was observed between loss of function *CYP2D6* alleles and increased risk for TD development in patients with schizophrenia treated with antipsychotics (Patsopoulos et al., 2005).

**TABLE 1:** Pharmacokinetics gene polymorphisms and associations with TD.

Gene	Genetic polymorphisms	Association	References
*CYP2D6*	CYP2D6*2	No	Ohmori et al. (1999); Inada et al. (2003); de Leon et al. (2005); Plesnicar et al. (2006); Grossman et al. (2008)
	CYP2D6*3	Yes	Kapitany et al. (1998); Jaanson et al. (2002); Ellingrod et al. (2002)
		No	Arthur et al. (1995); Armstrong et al. (1997); Andreassen et al. (1997); Ohmori et al. (1998); Scordo et al. (2000); Lohmann et al. (2003); Inada et al. (2003); de Leon et al. (2005); Plesnicar et al. (2006); Grossman et al. (2008); Ivanova et al. (2016a)
	CYP2D6*4	Yes	Kapitany et al. (1998); Jaanson et al. (2002); Ellingrod et al. (2002); Ivanova et al. (2016a)
		No	Arthur et al. (1995); Armstrong et al. (1997); Andreassen et al. (1997); Ohmori et al. (1998); Scordo et al. (2000); Lohmann et al. (2003); Inada et al. (2003); Tiwari et al. (2005b); de Leon et al. (2005); Plesnicar et al. (2006); Grossman et al. (2008)
	CYP2D6*5	Yes	Kapitany et al. (1998)
		No	Arthur et al. (1995); Armstrong et al. (1997); Andreassen et al. (1997); Scordo et al. (2000); Lohmann et al. (2003); de Leon et al. (2005); Plesnicar et al. (2006); Grossman et al. (2008)
	CYP2D6*6	No	Andreassen et al. (1997); Scordo et al. (2000); Lohmann et al. (2003); de Leon et al. (2005); Plesnicar et al. (2006); Grossman et al. (2008)
	CYP2D6*7	No	Andreassen et al. (1997); de Leon et al. (2005)
	CYP2D6*8	No	de Leon et al. (2005); Plesnicar et al. (2006)
	CYP2D6*9	No	de Leon et al. (2005); Plesnicar et al. (2006); Grossman et al. (2008)
	CYP2D6*10	Yes	Ohmori et al. (1998); Lam et al. (2001); Fu et al. (2006)
		No	Inada et al. (2003); Liou et al. (2004); de Leon et al. (2005); Plesnicar et al. (2006); Grossman et al. (2008)
	CYP2D6*11	No	de Leon et al. (2005); Plesnicar et al. (2006)
	CYP2D6*12	No	Inada et al. (2003); Plesnicar et al. (2006)
	CYP2D6*14	No	de Leon et al. (2005); Plesnicar et al. (2006)
	CYP2D6*15	No	Brockmöller et al. (2002); de Leon et al. (2005); Plesnicar et al. (2006)
	CYP2D6*17	No	Brockmöller et al. (2002); de Leon et al. (2005); Grossman et al. (2008)
	CYP2D6*18	No	de Leon et al. (2005)
	CYP2D6*19	No	de Leon et al. (2005)
	CYP2D6*20	No	de Leon et al. (2005)
	CYP2D6*25	No	de Leon et al. (2005)
	CYP2D6*26	No	de Leon et al. (2005)
	CYP2D6*29	No	de Leon et al. (2005); Grossman et al. (2008)
	CYP2D6*30	No	de Leon et al. (2005)
	CYP2D6*31	No	de Leon et al. (2005)
	CYP2D6*35	No	de Leon et al. (2005)
	CYP2D6*36	No	de Leon et al. (2005)
	CYP2D6*37	No	de Leon et al. (2005)
	CYP2D6*40	No	de Leon et al. (2005)
	CYP2D6*41	No	de Leon et al. (2005); Grossman et al. (2008)
	CYP2D6*43	No	de Leon et al. (2005)
	CYP2D6*45	No	de Leon et al. (2005)
	*CYP2D6 duplications*	No	Scordo et al. (2000); de Leon et al. (2005); Plesnicar et al. (2006)
*CYP1A2*	CYP1A2*1F	Yes	Basile et al. (2000); Fu et al. (2006); Ivanova et al. (2015); Ivanova et al. (2016a)
		No	Schulze et al. (2001); Chong et al. (2003b); Matsumoto et al. (2004a); Grossman et al. (2008)
	CYP1A2*1C	Yes	Tiwari et al. (2005a)
		No	Matsumoto et al. (2004a)
*CYP17A1*	rs743572	Yes	Segman et al. (2002a); Ivanova et al. (2014)
*CYP3A4*	CYP3A4*1B	No	Tiwari et al. (2005b)
*ABCB1*	rs1045642	No	de Leon et al. (2005); De Luca et al. (2009)
	rs1922242	No	De Luca et al. (2009)
	rs1045642, rs1922242	Yes–haplotype	De Luca et al. (2009)

Differences in CYP2D6*10 allele frequency were also reported in gender analysis studies. More specifically, the frequency of CYP2D6*10 was higher in female than in male patients with schizophrenia that experienced TD (Lam et al., 2001). Additionally, the risk for developing TD was higher in male carriers of a non-functional or partially functional allele, for instance CYP2D6*5, CYP2D6*10B, CYP2D6*14, and CYP2D6*41 than in wild type allele carriers (Nikoloff et al., 2002). Notably, smoking is another important variable, given that a study that considered interactions between genotypes and smoking status, indicated that most American smokers treated with antipsychotics and being carriers of the CYP2D6*1/*3, *4 genotypes had TD (Ellingrod et al., 2002).

The impact of *CYP1A2* genetic variability in TD also yielded conflicting results. No association was recorded between TD and CYP1A2*1F (Schulze et al., 2001; Chong et al., 2003b; Matsumoto et al., 2004a) or CYP1A2*1C (Matsumoto et al., 2004a). However, higher AIMS scores were reported in CYP1A2*1F CC homozygotes (Basile et al., 2000; Ivanova et al., 2015). Two additional studies confirmed the association of the CYP1A2*1F genotype with TD development (Fu et al., 2006; Ivanova et al., 2016a). Interestingly, carriers of CYP1A2*1C variant allele who were smokers and received only FGAs had increased severity of TD (Tiwari et al., 2005a).

Finally, it was reported that the *CYP17A1* rs743572 affects the development of TD since carriers of the CC genotype had a lower risk for TD occurrence (Ivanova et al., 2014). Additionally, the same single nucleotide polymorphism (SNP) was associated with the risk for abnormal orofacial and distal involuntary movements in schizophrenia patients (Segman et al., 2002a).

Among transporters important for pharmacokinetics of antipsychotics only the *ABCB1* was assessed. It was reported that there is no association between the *ABCB1* rs1045642 or rs1922242 and TD development (de Leon et al., 2005; De Luca et al., 2009). Notably, the haplotypes of rs1045642 and rs1922242 may play a role in the severity of TD, given that T–A and T-T haplotypes were associated with lower and higher AIMS scores, respectively (De Luca et al., 2009).

## 4 Pharmacogenomic Studies Focusing on the Association Between Tardive Dyskinesia and Genes Involved in the Pharmacodynamics

### 4.1 Dopamine Receptor Genes

Dopamine is a neurotransmitter that has an important role in human brain functions. Its dysregulation has been found to cause psychosis and mood disorders, as well as movement disorders, such as Parkinson’s disease and Huntington’s disease (Goode-Romero et al., 2020). A variety of commonly prescribed antipsychotic drugs, such as aripiprazole, cariprazine and brexpiprazole, are partial agonists of dopamine receptors. The involvement of dopamine receptors in TD development has received much attention over the years (Table 2) due to their crucial role in antipsychotics’ mechanism of action (Wang et al., 2018; Azorin and Simon 2019). All of the studies are presented in this chaptercan be found in the Supplementary Table S2.

**TABLE 2:** Genetic variability of dopamine receptors and its association with TD.

Genes	Genetic variations	Association	References
*DRD1*	rs4532	Yes	Lai et al. (2011b)
		No	Srivastava et al. (2006)
	rs5326	No	Lai et al. (2011b)
	rs265975	No	Lai et al. (2011b)
	rs5330	No	Srivastava et al. (2006)
	rs5331	No	Srivastava et al. (2006)
	rs13306309	No	Srivastava et al. (2006)
	rs686	No	Srivastava et al. (2006)
*DRD2*	rs1800497	Yes	Chen et al. (1997); Liou et al. (2006); Zai et al. (2007a); Bakker et al. (2008)
		No	Hori et al. (2001); Kaiser et al. (2002); Segman et al. (2003); Srivastava et al. (2006); Park et al. (2011); Koning et al. (2012); Lu et al. (2018)
	rs6275	Yes	Zai et al. (2007b)
		No	Park et al. (2011)
	rs6277	Yes	Zai et al. (2007b)
		No	Koning et al. (2012); Lu et al. (2018)
	rs1079597	Yes	Liou et al. (2006)
		No	Kaiser et al. (2002)
	rs1799732	No	Hori et al. (2001); Kaiser et al. (2002); Segman et al. (2003); de Leon et al. (2005); Srivastava et al. (2006); Liou et al. (2006); Zai et al. (2007b); Park et al. (2011); Koning et al. (2012)
	rs1799978	No	Kaiser et al. (2002)
	rs1800496	No	Kaiser et al. (2002); Zai et al. (2007b)
	rs1800497	No	Zai et al. (2007b)
	rs1800498	No	(Kaiser et al., 2002; Liou et al., 2006; Zai et al., 2007b; Park et al., 2011; Koning et al., 2012)
	rs1800499	No	Kaiser et al. (2002)
	rs1801028	No	Hori et al. (2001); Kaiser et al. (2002); Segman et al. (2003); Chong et al. (2003a); de Leon et al. (2005); Srivastava et al. (2006); Liou et al. (2006); Park et al. (2011)
	rs4648317	No	Zai et al. (2007b)
	rs1079598	No	Zai et al. (2007b)
	rs2242591	No	Zai et al. (2007b)
	rs2242592	No	Zai et al. (2007b)
	rs2242593	No	Zai et al. (2007b)
	Val96Ala	No	Kaiser et al. (2002)
	rs2234689	No	Srivastava et al. (2006)
	rs17294542	No	Srivastava et al. (2006)
	rs1125394	No	Zai et al. (2007b)
*DRD3*	rs6280	Yes	Steen et al. (1997); Segman et al. (1999); Segman et al. (2002a); Basile et al. (1999); Liao et al. (2001); Lerer et al. (2002); Woo et al. (2002); de Leon et al. (2005); Al Hadithy et al. (2009)
		No	Gaitonde et al. (1996); Rietschel et al. (2000); Løvlie et al. (2000); Segman et al., 2000; Garcia-Barceló et al. (2001); Chong et al. (2003a); Srivastava et al. (2006); Zai et al. (2009b); Utsunomiya et al., 2012; Koning et al. (2012)
	rs9817063	No	Bakker et al. (2012); Ivanova et al. (2012b)
	rs2134655	No	Zai et al. (2009b); Bakker et al. (2012); Ivanova et al. (2012b)
	rs963468	No	Bakker et al. (2012); Ivanova et al. (2012b)
	rs324035	No	(Bakker et al., 2012; Ivanova et al., 2012b)
	rs3773678	No	Bakker et al. (2012); Ivanova et al. (2012b)
	rs167771	No	Bakker et al. (2012); Ivanova et al. (2012b)
	rs11721264	No	Bakker et al. (2012); Ivanova et al. (2012b)
	rs167770	No	Zai et al. (2009b); Bakker et al. (2012); Ivanova et al. (2012b)
	rs7633291	No	Zai et al. (2009b); Bakker et al. (2012); Ivanova et al. (2012b)
	rs1800828	No	Bakker et al. (2012); Ivanova et al. (2012b)
	rs3732782	No	Zai et al. (2009b)
	rs905568	No	Srivastava et al. (2006); Zai et al. (2009b)
	rs7620754	No	Zai et al. (2009b)
	rs7616367	No	Zai et al. (2009b)
	rs7611535	No	Zai et al. (2009b)
	rs1394016	No	Zai et al. (2009b)
	rs9825563	No	Zai et al. (2009b)
	rs1800828	No	Zai et al. (2009b)
	rs2399496	No	Zai et al. (2009b)
	rs2087017	No	Zai et al. (2009b)
	rs1025398	No	Zai et al. (2009b)
	rs3732782, rs905568, rs7620754	Yes - haplotype	Zai et al. (2009b)
	rs324026	No	Srivastava et al. (2006)
	rs1503670	No	Srivastava et al. (2006)
	biallelic STR	No	Srivastava et al. (2006)
*DRD4*	120-bp tandem duplication	Yes	Srivastava et al. (2006)
		No	Segman et al. (2003)
	rs11246226	No	Zai et al. (2009a)
	48 bp VNTR exon 3	No	Segman et al. (2003); Srivastava et al. (2006); Zai et al. (2009a)
	rs936465	No	Zai et al. (2009a)
	rs3758653	No	Zai et al. (2009a); Bakker et al. (2012); Ivanova et al. (2012b)
	rs3758653, rs916457, rs762502, rs11246226	Yes–haplotype	Zai et al. (2009a)
	rs762502	No	Zai et al. (2009a)
	rs916457	No	Zai et al. (2009a)
	rs1800955	No	Srivastava et al. (2006)

D1 dopamine receptor is mainly found in the central nervous system. It regulates neuronal growth and development, and mediates behavioral and cognitive functions by stimulating adenylyl cyclase and activating cyclic AMP-dependent protein kinases (Dolzan et al., 2007). *DRD1* rs5326, rs4532 and rs265975 polymorphisms have been investigated as potential TD biomarkers. Schizophrenia patients with the *DRD1* rs4532 CC genotype had increased chance for TD development, while the rest of the studied polymorphisms did not reach statistical significance (Lai et al., 2011b). Nevertheless, the association between rs4532 and TD was not supported in a cohort of Indian patients with TD (Srivastava et al., 2006).

Dopamine receptor D2 protein inhibits adenylyl cyclase activity. Genetic variability of *DRD2* has been associated with schizophrenia and response to both FGAs and SGAs, as well as myoclonus dystonia (Klein et al., 1999; Wu et al., 2005; Fan et al., 2010; Wang et al., 2018). Studied *DRD2* polymorphisms which did not reach statistical significance in neither allelic nor genotypic level include rs1801028 (Hori et al., 2001; Kaiser et al., 2002; de Leon et al., 2005; Srivastava et al., 2006; Park et al., 2011), rs1800497 (Hori et al., 2001; Kaiser et al., 2002; Segman et al., 2003; Srivastava et al., 2006; Park et al., 2011; Koning et al., 2012; Lu et al., 2018), rs1799732 (Hori et al., 2001; Kaiser et al., 2002; Segman et al., 2003; de Leon et al., 2005; Liou et al., 2006; Srivastava et al., 2006; Zai et al., 2007b; Park et al., 2011; Koning et al., 2012), rs1801028 (Hori et al., 2001; Kaiser et al., 2002; Chong et al., 2003a; Segman et al., 2003; de Leon et al., 2005; Liou et al., 2006; Srivastava et al., 2006; Park et al., 2011), rs1799978, rs1079597, p. Val96Ala, rs1800499, rs1800496 (Kaiser et al., 2002), rs1800498 (Kaiser et al., 2002; Liou et al., 2006; Zai et al., 2007b; Park et al., 2011; Koning et al., 2012), rs2234689, and rs17294542 (Srivastava et al., 2006). However, an older study performed in patients with schizophrenia indicated that rs1800497 might affect the development of TD, given that the A2 allele and A2/A2 genotype were more frequent in female patients with TD (Chen et al., 1997). In addition, two meta-analyses and a comparative study suggested that the A2 allele and the A2/A2 genotype of rs1800497 was associated with increased TD risk (Liou et al., 2006; Zai et al., 2007a; Bakker et al., 2008). Regarding rs1079597, B2 allele and B2/B2 genotype has been associated with increased TD risk (Liou et al., 2006). A meta-analysis that included 1256 patients with schizophrenia concluded that rs1799732 was not associated with TD (Zai et al., 2007a). Rs6275 (Park et al., 2011) and rs6277 (Koning et al., 2012) gave negative results for potential association with TD. Nonetheless, the study of Zai et al. found that rs6277 T allele and rs6275 C allele frequencies were lower in TD patients as compared to patients with schizophrenia without TD (Zai et al., 2007b).

Dopamine D3 receptor is associated with cognitive, emotional, and endocrine functions due to its localization in the limbic areas of the brain. It signals through G proteins which inhibit adenylyl cyclase. Pathologies associated with *DRD3* polymorphisms include schizophrenia and hereditary essential tremor (Jeanneteau et al., 2006; Nunokawa et al., 2010; Sáiz et al., 2010). Many scientific groups focused on the association between *DRD3* rs6280 and TD, which gave conflicting results. Some studies indicated no association with TD development, on neither allelic nor genotypic level (Gaitonde et al., 1996; Løvlie et al., 2000; Rietschel et al., 2000; Segman et al., 2000; Garcia-Barceló et al., 2001; Chong et al., 2003a; Srivastava et al., 2006; Zai et al., 2009b; Koning et al., 2012; Utsunomiya et al., 2012). Several meta-analyses have also been conducted, which either observed a significant contribution of the p.9Gly allele to the higher risk for TD (Lerer et al., 2002; Bakker et al., 2006) or failed to prove a significant association (Tsai et al., 2010). There was no association on the genotypic level, probably due to the small effect size and ethnic diversity. The authors concluded that *DRD3* rs6280 might be associated with TD, but caution is required in interpreting this result (Bakker et al., 2006). However, some studies showed a correlation between TD with either the p.9Gly allele (Steen et al., 1997; Lerer et al., 2002; Bakker et al., 2006), or p.9Gly homozygotes (Basile et al., 1999; Woo et al., 2002) and heterozygotes (Liao et al., 2001). Additionally, *DRD3* rs6280 was associated with a subtype of TD, namely limb-truncal dyskinesia (Al Hadithy et al., 2009). Other *DRD3* polymorphisms that have been studied but indicated no association with TD include rs3732782, rs905568, rs7620754, rs7616367, rs7611535, rs1394016, rs9825563, rs1800828, rs2399496, rs2087017, rs1025398, rs9817063, rs2134655, rs963468, rs324035, rs3773678, rs167771, rs11721264, rs167770, rs7633291 and rs1800828 (Zai et al., 2009b; Bakker et al., 2012; Ivanova et al., 2012b) rs324026, rs1503670 and biallelic STR (Srivastava et al., 2006). However, it is important to mention that the study of Zai et al. and his colleagues reported an association between the *DRD3* haplotype of rs3732782, rs905568, and rs7620754 and TD and they provided evidence of interaction between *BDNF* and *DRD3* polymorphisms.

Finally, *DRD4* rs3758653, rs11246226, 48 bp VNTR exon 3, rs936465, rs3758653, rs762502, rs916457, rs1800955 have been investigated in connection to TD, but did not show any correlation (Segman et al., 2003; Srivastava et al., 2006; Zai et al., 2009a; Bakker et al., 2012; Ivanova et al., 2012b).

However, association between the haplotype of rs3758653, rs916457, rs762502, rs11246226 and TD was reported (Zai et al., 2009a), as well as association between the 120 bp duplication marker in *DRD4* and TD in genotypic level (Srivastava et al., 2006), which was not replicated (Segman et al., 2003). All of the studies are presented in this chaptercan be found in the Supplementary Table S2.

### 4.2 Serotonin Receptor Genes

Serotonin is a neurotransmitter, which plays a crucial role in brain pathways. Members of the 5-hydroxytryptamine receptor subfamily encode seven different receptors for serotonin. HTR2A, HTR2B and HTR2C are positively coupled with the phospholipase C enzyme (PLC) and have been investigated for their potential involvement in TD in many different populations (Table 3).

**TABLE 3:** Genetic variability of serotonin receptors and its association with TD.

Genes	Genetic variations	Association	References
*HTR1A*	rs6295	No	Pozhidaev et al. (2020)
	rs1364043	No	Pozhidaev et al. (2020)
	rs10042486	No	Pozhidaev et al. (2020)
	rs1800042	No	Pozhidaev et al. (2020)
	rs749099	No	Pozhidaev et al. (2020)
*HTR1B*	rs6298	No	Pozhidaev et al. (2020)
	rs6296	No	Pozhidaev et al. (2020)
	rs130058	No	Pozhidaev et al. (2020)
*HTR2A*	rs1928040	Yes	Pozhidaev et al. (2020)
	rs6311	Yes	Segman et al. (2001); Boke et al. (2007)
		No	Basile et al. (2001); Herken et al. (2003); Al Hadithy et al. (2009); Pozhidaev et al. (2020)
	rs6313	Yes	Segman et al. (2001); Tan et al. (2001); Hsieh et al. (2011)
		No	Basile et al. (2001); Herken et al. (2003); Koning et al. (2012); Pozhidaev et al. (2020)
	rs6314	No	Basile et al. (2001); Segman et al. (2001); Koning et al. (2012); Pozhidaev et al. (2020)
	rs7997012	No	Pozhidaev et al. (2020)
	rs9316233	No	Pozhidaev et al. (2020)
	rs2224721	No	Pozhidaev et al. (2020)
*HTR2C*	rs518147	Yes	Zhang et al. (2002a)
	rs6318	Yes	Segman et al. (2000); Al Hadithy et al. (2009)
		No	Hsieh et al. (2011); Ivanova et al. (2012b); Koning et al. (2012); Pozhidaev et al. (2020)
	rs1801412	Yes	Pozhidaev et al. (2020)
		No	Bakker et al. (2012); Pozhidaev et al. (2020)
	rs4911871	No	Bakker et al. (2012)
	rs12858300	No	Bakker et al. (2012); Pozhidaev et al. (2020)
	rs17326429	No	Bakker et al. (2012); Pozhidaev et al. (2020)
	rs3813929	No	Zhang et al. (2002a); Ivanova et al. (2012b); Koning et al. (2012); Pozhidaev et al. (2020)
	rs4911871	No	Ivanova et al. (2012b); Pozhidaev et al. (2020)
	rs569959	No	Bakker et al. (2012); Ivanova et al. (2012b); Pozhidaev et al. (2020)
	rs5946189	No	Bakker et al. (2012); Pozhidaev et al. (2020)
*HTR3A*	rs1062613	No	Kang et al. (2013); Pozhidaev et al. (2020)
	rs33940208	No	Pozhidaev et al. (2020)
	rs1176713	No	Pozhidaev et al. (2020)
*HTR3B*	rs1176744	No	Pozhidaev et al. (2020)
*HTR6*	rs1805054	No	Ohmori et al. (2002); Segman et al. (2003); Pozhidaev et al. (2020)

5-HT2A receptors are postsynaptic receptors that regulate the function of prefrontal-subcortical circuits. The 5-HT2A receptor interacts with G proteins and stimulates PLC to produce the intracellular second messengers sn-1,2-DAG and inositol-1,4,5-trisphosphate (IP3), which control the calcium channel (McMahon et al., 2006). Mutations in the *HTR2A* gene were shown to be associated with susceptibility to mental disorders, such as schizophrenia, depression and obsessive-compulsive disorder, alcohol dependence and response to antidepressants (Williams et al., 1996; McMahon et al., 2006; Yasseen et al., 2010). *HTR2A* rs6313, rs6311 and rs6314 have been extensively studied for their potential association with TD development. According to some studies, rs6313, rs6311 and rs6314 did not reach statistical significance (Basile et al., 2001; Herken et al., 2003; Koning et al., 2012). However, *HTR2A* rs6313 T allele was associated with TD development (Tan et al., 2001; Hsieh et al., 2011), but on the contrary the frequency of *HTR2A* rs6313 TT homozygotes was higher in patients without TD (Tan et al., 2001). Additionally, it has been reported that the frequencies of *HTR2A* rs6313 C allele and *HTR2A* rs6311 G allele were higher in patients with TD. In line with that, the *HTR2A* rs6313 CC and *HTR2A* rs6311 GG (rs6311) genotypes were associated with higher AIMS scores (Segman et al., 2001) and rs6311 presented a trend of association with TD in Turkish patients with schizophrenia under prolonged exposure to antipsychotics (Boke et al., 2007). Additionally, the *HTR2A* rs6313 CC genotype was associated with TD development in older patients and those with limb-truncal TD (Lerer et al., 2005). The haplotype analysis of the *HTR2A* rs6313 and *HTR2A* rs6314 showed significant associations with TD (Lerer et al., 2005). Finally, *HTR2A* rs1928040 was associated with the orofacial type of TD (Pozhidaev et al., 2020).

Regarding *HTR2C* genetic variability rs6318 has been thoroughly studied over the years. Two studies indicated no association with TD (Hsieh et al., 2011; Koning et al., 2012). However, a study published in 2000 showed that the frequency of the Ser allele was more frequent in patients with TD (Segman et al., 2000). Additionally, *HTR2C* rs6318 was associated with limb-truncal but not with orofaciolingual dyskinesia in the Russian population (Al Hadithy et al., 2009). Another studied polymorphism of *HTR2C* rs518147 was more frequent in patients with schizophrenia that experienced TD (Zhang et al., 2002a; Koning et al., 2012). Other *HTR2C* polymorphisms were studied, such as rs569959, rs17326429, rs12858300, rs4911871, rs5946189, rs1801412 (Bakker et al., 2012), and rs3813929 (Zhang et al., 2002a; Koning et al., 2012), but no association with TD was recorded. However, *HTR2C* rs1801412 was significantly associated with the orofacial type of TD in women (Pozhidaev et al., 2020).

Lastly, there are three studies that focused on *HTR6* rs1805054. No association with TD was reported in Japanese, Ashkenazi, non-Ashkenazi and Caucasian schizophrenia patients (Ohmori et al., 2002; Segman et al., 2003; Pozhidaev et al., 2020). All of the studies are presented in this chaptercan be found in the Supplementary Table S2.

### 4.3 Other Neurotransmission Receptors

Several other neurotransmitters and their receptors may also play a role in the development of TD (Table 4).

**TABLE 4:** Genetic variability of other neurotransmitter receptors and their association with TD.

Genes	Genetic variations	Association	References
*CHRM1*	rs2075748	No	Boiko et al. (2020)
	rs544978	No	Boiko et al. (2020)
	rs2067477	No	Boiko et al. (2020)
	rs2067479	No	Boiko et al. (2020)
	rs2186410	No	Boiko et al. (2020)
	rs542269	No	Boiko et al. (2020)
*CHRM2*	rs2061174	Yes	Boiko et al. (2020)
	rs1824024	Yes	Boiko et al. (2020)
	rs324650	No	Boiko et al. (2020)
	rs2350780	No	Boiko et al. (2020)
	rs7810473	No	Boiko et al. (2020)
	rs2350786	No	Boiko et al. (2020)
	rs324640	No	Boiko et al. (2020)
	rs1378650	No	Boiko et al. (2020)
*CNR1*	rs806374	Yes	Tiwari et al. (2012)
	rs12720071	No	Tiwari et al. (2012)
	rs1049353	No	Tiwari et al. (2012)
	rs80639	No	Tiwari et al. (2012)
	rs806370	No	Tiwari et al. (2012)
	rs806368	No	Tiwari et al. (2012)
	rs806375	No	Tiwari et al. (2012)
	rs806377	No	Tiwari et al. (2012)
	rs806378	No	Tiwari et al. (2012)
	rs2023239	No	Tiwari et al. (2012)
	rs806380	No	Tiwari et al. (2012)
	rs806381	No	Tiwari et al. (2012)
	rs7752758	No	Tiwari et al. (2012)
	rs12528858	No	Tiwari et al. (2012)
	rs12205430	No	Tiwari et al. (2012)
	rs6914429	No	Tiwari et al. (2012)
	rs2180619	No	Tiwari et al. (2012)
	rs754387	No	Tiwari et al. (2012)
	rs9450902	No	Tiwari et al. (2012)
	rs10485170	No	Tiwari et al. (2012)
*GABRB2*	rs918528	No	Son et al. (2014)
*GABRG3*	rs2061051	No	Son et al. (2014)
*GRIN2A*	rs7206256	Yes	Ivanova et al. (2012b)
	rs1345423	Yes	Ivanova et al. (2012b); Ivanova et al. (2016b)
		No	Bakker et al. (2012)
	rs7190619	Yes	Ivanova et al. (2012b)
		No	Bakker et al. (2012)
	rs9788936	Yes	Ivanova et al. (2012b)
		No	Bakker et al. (2012)
	rs11646587	Yes	Ivanova et al. (2012b)
		No	Bakker et al. (2012)
	rs9921541	No	Bakker et al. (2012); Ivanova et al. (2012b)
	rs7192557	No	Bakker et al. (2012); Ivanova et al. (2012b)
	rs8049651	No	Bakker et al. (2012); Ivanova et al. (2012b)
	rs7196095	No	Bakker et al. (2012); Ivanova et al. (2012b)
	rs11866328	No	Bakker et al. (2012); Ivanova et al. (2012b)
	rs4782039	No	Bakker et al. (2012); Ivanova et al. (2012b)
	rs11644461	No	Bakker et al. (2012); Ivanova et al. (2012b)
	rs9989388	No	Bakker et al. (2012); Ivanova et al. (2012b)
	rs8057394	No	Bakker et al. (2012); Ivanova et al. (2012b)
	rs1650420	No	Bakker et al. (2012); Ivanova et al. (2012b)
*GRIN2B*	rs2192970	Yes	Ivanova et al. (2012b)
		No	Bakker et al. (2012)
	rs1805481	No	Ivanova et al. (2012b)
	rs7313149	No	Bakker et al. (2012); Ivanova et al. (2012b)
	rs2300242	No	Bakker et al. (2012); Ivanova et al. (2012b)
	rs10845838	No	Bakker et al. (2012); Ivanova et al. (2012b)
	rs12300851	No	Bakker et al. (2012); Ivanova et al. (2012b)
	rs220599	No	Bakker et al. (2012); Ivanova et al. (2012b)
	rs10772715	No	Bakker et al. (2012); Ivanova et al. (2012b)
	rs12827536	No	Bakker et al. (2012); Ivanova et al. (2012b)
*OPRD1*	921 T/C	No	Ohmori et al. (2001)
*OPRM1*	rs1799971	Yes	Ohmori et al. (2001)

The glutamate system has also been examined for potential involvement in TD pathophysiology. *GRIN2A* encodes the N-methyl-d-aspartate (NMDA) receptor, which belongs to the glutamate-gated ion channel family. These receptors are involved in crucial signaling pathways, and their dysregulation is associated with epilepsy, cognitive and speech deficits, and intellectual disability (Endele et al., 2010; Lemke et al., 2013; Lesca et al., 2013). Three studies have investigated the potential role of NMDA glutamate receptor genes, *GRIN2A* and *GRIN2B* in TD (Bakker et al., 2012; Ivanova et al., 2012b; Ivanova et al., 2016b). Except for *GRIN2A* rs7206256, which was associated with orofacial TD (Ivanova et al., 2012b), conflicting results emerged for *GRIN2A* rs1345423, rs7190619, rs9788936 and rs11646587 (Bakker et al., 2012; Ivanova et al., 2012b; Ivanova et al., 2016b). The same stands for *GRIN2B* rs2192970 (Bakker et al., 2012; Ivanova et al., 2012b), while the rest of the studied polymorphisms were not associated with TD (Bakker et al., 2012; Ivanova et al., 2012b; Ivanova et al., 2016b). The role of GABA receptor genes *GABRB2* and *GABRG3* in TD has been investigated, but no significant associations were reported for the studied polymorphisms. However, gene-gene interactions between *GABRB2*, *GABRG3*, and *SCL6A11* and susceptibility to TD highlighted the importance of the GABA receptor signaling pathway (Son et al., 2014).

Muscarinic receptors participate in various cellular responses such as inhibition of adenylate cyclase, degeneration of phosphoinositide, and control of potassium channel. These receptors can influence the function of the central and peripheral nervous system through acetylcholine-induced signaling (Luo et al., 2005). Blocking muscarinic receptors with anticholinergic drugs is a common therapeutic approach for treatment of the drug-induced parkinsonism and dystonia. Assessment of the association of muscarinic cholinergic receptor 1 (CHRM1) and 2 (CHRM2) genetic variability with TD development showed a trend towards *CHRM2* rs2061174 and rs1824024 effect on TD risk (Boiko et al., 2020). Interestingly, CHRM2 is involved in neurophysiological processes, mood disorders, alcohol and nicotine abuse (Luo et al., 2005; Dick et al., 2007; Mobascher et al., 2010).

Opioid receptors can act as dopamine regulators and target endogenous opioid peptides and analgesics. Genetic variability of opioid receptors has been associated with substance abuse and addiction, as well as schizophrenia (Deb et al., 2010; Serý et al., 2010). Genetic variability of *OPRD1* indicated no association with TD. However, *OPRM1* rs1799971 showed to be a promising biomarker of TD development since the frequency of the G allele was lower in patients with TD (Ohmori et al., 2001).

Cannabinoid receptors are members of the G-protein coupled receptor family, which inhibits adenylate cyclase activity in a dose-dependent way. Cannabinoid use induces symptoms, such as anxiety, memory loss and chronic pain, which is associated with cannabinoid receptors 1 (CNR1) and 2 (CNR2) (Oddi et al., 2012; Hua et al., 2016). Apart from their association with cannabis dependence and abuse, and heroin addiction, cannabinoid receptors have also been related to antipsychotic-induced weight gain in patients with schizophrenia (Proudnikov et al., 2010; Tiwari et al., 2010; Arias Horcajadas et al., 2021). The potential role of CNR1 as an activator in movement inhibition has also been investigated. The findings indicate an association between the *CNR1* rs806374 CC genotype with both the development and severity of TD. However, more studies are needed to replicate this association (Tiwari et al., 2012).All of the studies are presented in this chaptercan be found in the Supplementary Table S2.

### 4.4 Neurotransmitter Transporters

*SLC18A2* encodes the vesicular monoamine transporter 2 protein, a transmembrane protein that regulates neurotransmission through transportation of monoamines, such as dopamine, serotonin, and norepinephrine, into the intracellular vesicles. Psychiatric disorders like schizophrenia, as well as movement disorders (Rilstone et al., 2013), nicotine and alcohol dependence (Schwab et al., 2005) and antidepressant response (Crowley et al., 2008) have been associated with *SLC18A2* polymorphisms. *SLC18A2* rs363390, rs14240, rs1860404, rs2015586 (Zai et al., 2013), and rs363224 were all associated with TD development (Zai et al., 2013; Lu et al., 2018).

*SLC6A4* encodes the serotonin transporter (5-HTT), a membrane protein that transports serotonin from the synaptic cleft back to presynaptic neurons. Genetic variability of *SLC6A4* is associated with response to antidepressant and lithium treatment (Rybakowski et al., 2009; Tansey et al., 2012). However, studies focusing on *SLC6A4* and TD development provided no evidence of association (Chong et al., 2000; Herken et al., 2003; Segman et al., 2003; Hsieh et al., 2011).

*SLC6A3* encodes a dopamine transporter, member of the sodium- and chloride-dependent neurotransmitter transporter family. Variation in the number of repeats has been associated with attention-deficit hyperactivity disorder, alcohol and cocaine dependence, as well as susceptibility to Parkinson's disease (Lott et al., 2005; Kurian et al., 2009, 2011; Müller et al., 2010). The 40 bp tandem repeat VNTR found in the 3’ UTR was not associated with TD (Segman et al., 2003; Srivastava et al., 2006), and so was not the G2319A transversion either (Segman et al., 2003).

The protein encoded by *SLC6A11* is a sodium-dependent transporter of GABA from synaptic cleft to surrounding glial cells leading to decreased GABA signaling. A case-control study, which included 180 patients with schizophrenia, 105 of whom experienced TD, identified an association between *SLC6A11* rs4684742 and TD (Son et al., 2014) (Summarized in Table 5). All of the studies are presented in this chaptercan be found in the Supplementary Table S2.

**TABLE 5:** Genetic variability of neurotransmitter transporters and its associations with TD.

Genes	Genetic variations	Association	References
*SLC18A2*	rs363390	Yes	Zai et al. (2013)
	rs14240	Yes	Zai et al. (2013)
	rs1860404	Yes	Zai et al. (2013)
	rs2015586	Yes	Zai et al. (2013)
	rs363224	Yes	Zai et al. (2013); Lu et al. (2018)
	rs363285	No	Zai et al. (2013)
	rs363393	No	Zai et al. (2013)
	rs2072362	No	Zai et al. (2013)
	rs2244249	No	Zai et al. (2013)
*SLC6A4*	5-HTTLPR VNTR (5-HTT)	No	Chong et al. (2000); Herken et al. (2003); Segman et al. (2003); Hsieh et al. (2011)
*SLC6A3*	40 bp VNTR	No	Segman et al. (2003); Srivastava et al. (2006)
	*G2319A*	No	Segman et al. (2003)
*SLC6A11*	rs4684742	Yes	Son et al. (2014)

### 4.5 Neurotransmitter Biosynthesis and Degradation Pathways

Genes involved in neurotransmitter biosynthesis and degradation have also been studied for their potential role in the development and severity of TD (Table 6). However, only rs4680 located on the catechol-O-methyltransferase gene (*COMT*) indicated potential association, given that AA and AG genotypes were associated with decreased TD risk in comparison with carriers of GG genotype (Srivastava et al., 2006; Bakker et al., 2008). However, additional studies failed to replicate this result (Herken et al., 2003; Matsumoto et al., 2004a; Lai et al., 2005; Zai et al., 2010b; Koning et al., 2012; Li et al., 2013; Lv et al., 2016). The AA genotype of *COMT* rs165599 was associated with TD and a trend of association with higher AIMS scores was also reported in a mixed cohort of Caucasians and African Americans (Zai et al., 2010b). Allelic and genotypic associations were also recorded for rs4818 in a cohort of Indian patients with schizophrenia (Srivastava et al., 2006), which was not replicated in a later study (Zai et al., 2010b) (summarized in Table 6).

**TABLE 6:** Genetic variability of neurotransmitter biosynthesis and degradation pathways and its association with TD.

Genes examined	Genetic variations	Association	References
*COMT*	rs4680	Yes	Srivastava et al. (2006); Bakker et al. (2008)
		No	Herken et al. (2003); Matsumoto et al. (2004a); Lai et al. (2005); Zai et al. (2010b); Koning et al. (2012); Li et al. (2013); Lv et al. (2016)
	rs4818	Yes	Srivastava et al. (2006)
		No	Zai et al. (2010b)
	rs165599	Yes	Zai et al. (2010b)
	rs737865	No	Zai et al. (2010b)
	rs6269	No	Zai et al. (2010b)
	rs4633	No	Srivastava et al. (2006); Zai et al. (2010b)
	rs2075507	No	Srivastava et al. (2006)
	900 ins C 3 UTR	No	Srivastava et al. (2006)
*DBH*	rs72393728	No	Sun et al. (2013); Hui et al. (2015); Hui et al. (2017)
*MAOA*	30-bp repeat in the promoter region	No	Matsumoto et al. (2004b); Li et al. (2013)
*MAOB*	rs1799836	No	Matsumoto et al. (2004b)

Other studied genes of this category that did not reach statistical significance, include *DBH* coding for dopamine beta-hydroxylase (Sun et al., 2013; Zhou et al., 2013; Hui et al., 2015, 2017), and genes coding for monoamine oxidases, *MAOA* (Matsumoto et al., 2004b; Li et al., 2013) and *MAOB* (Matsumoto et al., 2004b).All of the studies are presented in this chaptercan be found in the Supplementary Table S2.

### 4.6 Developmental/Plasticity Factors

ErbB4 is a tyrosin kinase receptor and among others binds neuregulins and other compounds. It plays an essential role in neurotransmission and cellular responses. It is a regulator of GABA and dopamine signaling and mediates neuroplasticity (Vullhorst et al., 2009). Genetic variability of *ERBB4* has been associated with cancer, schizophrenia, and amyotrophic lateral sclerosis (Tvorogov et al., 2009; So et al., 2010; Takahashi et al., 2013). Regarding TD, *ERBB4* rs839523 CC genotype was associated with a high risk of developing TD and a higher chance for severe TD in a cohort of 153 European patients with schizophrenia (Zai et al., 2019a).

The gene coding for the Protein Regulating Synaptic Membrane Exocytosis 2 (*RIMS2*) has also been studied, and an association between rs567070433 and TD occurrence was observed (Alkelai et al., 2019). *RIMS2* encodes a protein that binds other proteins involved in neurotransmitter diffusion. Polymorphisms in this gene have been associated with degenerative lumbar scoliosis, congenital cone-rod synaptic disorder, neurodevelopmental disease, and abnormal glucose homeostasis in the elderly (Mechaussier et al., 2020).

Other genes that are involved in developmental plasticity, including *BDNF* (Zai et al., 2009b; Wang et al., 2010; Zhang et al., 2012), *DISC1* (Lu et al., 2018), NRXN1 (Lanning et al., 2017), and *NRG1* (Zai et al., 2019a) were investigated but showed no association with TD (Table 7). All of the studies are presented in this chaptercan be found in the Supplementary Table S2.

**TABLE 7:** Developmental and plasticity: genetic variations and associations with TD.

Genes	Genetic variations	Association	References
*ERBB4*	rs839523	Yes	Zai et al. (2019a)
*RIMS2*	rs567070433	Yes	Alkelai et al. (2019)
*NRXN1*	rs17041112	No	Lanning et al. (2017)
	rs10490162	No	Lanning et al. (2017)
	rs1400882	No	Lanning et al. (2017)
	rs12467557	No	Lanning et al. (2017)
	rs1045881	No	Lanning et al. (2017)
*NRG1*	rs35753505	No	Zai et al. (2019a)
	rs6994992	No	Zai et al. (2019a)
*BDNF*	rs6265	No	Zai et al. (2009b); Wang et al. (2010); Zhang et al. (2012)
	rs7934165	No	Zai et al. (2009b)
	rs11030104	No	Zai et al. (2009b)
	rs1519480	No	Zai et al. (2009b)
*DISC1*	rs2492367	No	Lu et al. (2018)
	rs3738398	No	Lu et al. (2018)
	rs1322784	No	Lu et al. (2018)
	rs11122359	No	Lu et al. (2018)
	rs821597	No	Lu et al. (2018)
	rs701158	No	Lu et al. (2018)
	rs3738401	No	Lu et al. (2018)
	rs6675281	No	Lu et al. (2018)
	rs821616	No	Lu et al. (2018)

### 4.7 Oxidative Stress-Related Genes

The role of oxidative stress-related genes has received the attention of various research groups over the years (Table 8). SOD2 belongs to the iron/manganese superoxide dismutase family and *SOD2* encodes a mitochondrial protein that binds manganese ions and products of oxidative phosphorylation. *SOD2* rs4880 is one of the most frequently studied polymorphisms of this gene, but conflicting results regarding its association with TD have been reported so far. Most of the studies indicated no association with TD (Zhang et al., 2002b; Zai et al., 2010a; Ivanova et al., 2012b; Koning et al., 2012), including a meta-analysis with a validation cohort of 223 patients with schizophrenia of Caucasian and African American ancestry (Zai et al., 2010a). However, an association between lower *SOD2* rs4880 Ala allele frequencies in TD affected patients was recorded (Hori et al., 2000), along with a significant difference in genotypic distribution between patients with and without TD (Hori et al., 2000; Hitzeroth et al., 2007).

**TABLE 8:** Oxidative stress pathway: genetic variations and associations with TD.

Genes	Genetic variations	Association	References
*GSTM1*	*GSTM1* deletion	Yes	de Leon et al. (2005)
*SOD2*	rs4880	Yes	Hori et al. (2000); Hitzeroth et al. (2007)
		No	Zhang et al. (2002b); Zai et al. (2010a); Ivanova et al. (2012b); Koning et al. (2012); Bošković et al. (2013)
*NOS1*	C/T polymorphism in exon 29	No	Shinkai et al. (2004)
*NQO1*	rs1800566	No	Zai et al. (2010a); Bakker et al. (2012); Ivanova et al. (2012b); Koning et al. (2012)
*GSK3B*	rs334558	No	Levchenko et al. (2019)
*GPX1*	rs1050450	No	Shinkai et al. (2006); Bošković et al. (2013)
*CAT*	rs1001179	No	Bošković et al. (2013)
	rs10836235	No	Bošković et al. (2013)
*GSTT1*	*GSTT1* deletion	No	de Leon et al. (2005)
*GSTP1*	rs1695	No	Shinkai et al. (2005); Koning et al. (2012)

*GSTM1* encodes a glutathione S-transferase mu 1. This enzyme conjugates with glutathione and detoxifies many electrophilic compounds, such as products of oxidative stress and drugs, influencing their toxicity and efficacy. Genetic variability of *GSTM1* is associated with cancer, including brain tumors and chronic diseases, like asthma (Schwartzbaum et al., 2007; Sakoda et al., 2008; Nguyen et al., 2010; Piacentini et al., 2010). Association between *GSTM1* deletion and TD development has also been observed in Caucasians taking risperidone, olanzapine, quetiapine or FGAs (de Leon et al., 2005).

Other oxidative stress-related genes were also studied but indicated no association with TD. These genes include *NOS1* (Shinkai et al., 2004), *NQO1* (Zai et al., 2010a; Bakker et al., 2012; Ivanova et al., 2012b; Koning et al., 2012), *GSK3*B (Levchenko et al., 2019), *GPX1* (Shinkai et al., 2006; Bošković et al., 2013), *CAT* (Bošković et al., 2013), *GSTT1* (de Leon et al., 2005), and *GSTP1* (Shinkai et al., 2005; Koning et al., 2012). All of the studies are presented in this chaptercan be found in the Supplementary Table S2.

### 4.8 Inflammation and Tardive Dyskinesia

Genes involved in inflammation have also been proposed to be associated with TD (Table 9). Genes that encode proteins involved in inflammation, such as *IL10* (Sun et al., 2013), and *TNF* (Wang et al., 2012; Bošković et al., 2013) were studied and showed no association with TD.

**TABLE 9:** Inflammation: genetic variations and associations with TD.

Genes	Genetic variations	Association	References
*IL10*	rs1800872	No	Sun et al. (2013)
*TNF*	rs1800629	No	Wang et al. (2012); Bošković et al. (2013)
*C4A*	*C4AL*	No	Zai et al. (2019b)
	*C4AS*	No	Zai et al. (2019b)
	*C4A*	No	Zai et al. (2019b)
*C4B*	*C4BL*	Yes	Zai et al. (2019b)
	*C4BS*	No	Zai et al. (2019b)
	*C4B*	No	Zai et al. (2019b)

Complement component 4 (C4) is part of the complement system which mediates immunity. C4 was implicated in schizophrenia pathophysiology and motor movement after injury. The investigation of copy number variations of the long (L) and short (S) forms of *C4A* and *C4B* in Europeans has shown a nominally significant association between *C4BL* and TD severity (Zai et al., 2019b). All of the studies are presented in this chaptercan be found in the Supplementary Table S2.

### 4.9 Other Genes

Other studied candidate genes and their polymorphisms have been investigated for potential association with TD (Table 10).

**TABLE 10:** Genetic variability of other genes and associations with TD.

Genes	Genetic variations	Association	References
*PIP4K2A*	rs10828317	Yes	Fedorenko et al. (2014)
	rs746203	No	Fedorenko et al. (2014)
	rs8341	No	Fedorenko et al. (2014)
*ESR1*	NA	Yes	Lai et al. (2002)
*ApoE*	ApoE ε2	No	Kimura et al. (2000)
	ApoE ε3	No	Kimura et al. (2000)
	ApoE ε4	No	Kimura et al. (2000)
*PAWR*	rs7979987	No	Kim et al. (2012)
	rs4842318	No	Kim et al. (2012)
	rs17005769	No	Kim et al. (2012)
*DTNBP1*	rs760761	No	Maes et al. (2021)
	rs3213207	No	Maes et al. (2021)
	rs16876738	No	Maes et al. (2021)
	rs2619539	No	Maes et al. (2021)
	rs875462	No	Maes et al. (2021)
	rs17470454	No	Maes et al. (2021)
	rs2619539, rs875462, rs17470454	Yes - haplotype	Maes et al. (2021)
	rs760761, rs3213207, rs16876738	Yes - haplotype	Maes et al. (2021)
*MTNR1A*	rs11721818	No	Lai et al. (2011a)
	rs2375801	No	Lai et al. (2011a)
	rs6553010	No	Lai et al. (2011a)
	rs11721818, rs2375801, rs6553010	Yes - haplotype	Lai et al. (2011a)
*MTNR1B*	rs4753426	No	Lai et al. (2011a)
	rs10830963	No	Lai et al. (2011a)
	rs3781637	No	Lai et al. (2011a)
*ADORA1*	rs1874142	No	Turčin et al. (2016)
	rs10920568	No	Turčin et al. (2016)
	rs3766566	No	Turčin et al. (2016)
	rs3766560	No	Turčin et al. (2016)
	rs3753472	No	Turčin et al. (2016)
	rs3766553	No	Turčin et al. (2016)
	rs12744240	No	Turčin et al. (2016)
*ADORA2A*	rs35060421	No	Ivanova et al. (2012a)
	rs2298383	No	Turčin et al. (2016)
	rs17004921	No	Turčin et al. (2016)
	rs5751876	No	Turčin et al. (2016)
	rs35320474	No	Turčin et al. (2016)
	rs2236624	No	Turčin et al. (2016)
*ADORA3*	rs3394	No	Turčin et al. (2016)
	rs3393	No	Turčin et al. (2016)
	rs2229155	No	Turčin et al. (2016)
	rs35511654	No	Turčin et al. (2016)
	rs1544223	No	Turčin et al. (2016)
	rs2298191	No	Turčin et al. (2016)
	rs3394, rs3393, rs2229155, rs35511654, rs1544223, rs2298191	Yes - haplotype	Turčin et al. (2016)
*ACE*	insertion/deletion in the 16th intron	No	Segman et al. (2002b)
*AKT1*	rs3730358	No	Zai et al. (2008); Levchenko et al. (2019)
	rs1130214	No	Zai et al. (2008); Levchenko et al. (2019)
	rs2498784	No	Zai et al. (2008)
	rs2494746	No	Zai et al. (2008)
	rs10149779	No	Zai et al. (2008)
	rs2494738	No	Zai et al. (2008)
	rs3803304	No	Zai et al. (2008)
	rs2494731	No	Zai et al. (2008)
*HSPG2*	rs2445142	Yes	Greenbaum et al. (2012); Zai et al. (2018a)
		No	Bakker et al. (2012)
	rs2270697	No	Bakker et al. (2012); Ivanova et al. (2012b)
	rrs4738269	No	Greenbaum et al. (2012)
	rs2061051	No	Greenbaum et al. (2012)
	rs2124368	No	Greenbaum et al. (2012)
	rs6698486	No	Bakker et al. (2012)
	rs886292	No	Greenbaum et al. (2012)
*RGS2*	rs4606	No	Koning et al. (2012)
TPH	rs1800532	No	Segman et al. (2003)

Adenosine receptors are involved in many intracellular signaling pathways as homeostatic modulators of adenosine in the central nervous system. Even though there were no associations between polymorphisms of *ADORA1, ADORA2A*, and *ADORA3* and TD (Ivanova et al., 2012a; Turčin et al., 2016), it should be mentioned that the *ADORA3* CACTAT haplotype of rs3394, rs3393, rs2229155, rs35511654, rs1544223, and rs2298191 polymorphisms was associated with TD (Turčin et al., 2016).

*HSPG2* encodes perlecan, a proteoglycan that binds components of the matrix and cell membrane and is involved in essential biological processes. Perlecan participates in vascularization and maintains endothelial barrier function and vascular homeostasis. Genetic variability of *HSPG2* has been associated with skeletal disorders (Arikawa-Hirasawa et al., 2001, Arikawa-Hirasawa et al., 2002). *HSPG2* polymorphisms were not associated with TD in several different studies (Bakker et al., 2012; Ivanova et al., 2012b; Greenbaum et al., 2012). However, the assessment of *HSPG2* rs2445142 in association with TD returned both non-significant (Bakker et al., 2012) and significant results (Greenbaum et al., 2012; Zai et al., 2018a). A meta-analysis of 324 patients with TD and 515 without TD indicated that *HSPG2* rs2445142 G allele was significantly associated with TD (Zai et al., 2018a). Association between *HSPG2* rs2445142 G allele and TD risk was also reported in a patient cohort of Jewish Israeli descent (Greenbaum et al., 2012).

Even though studied polymorphisms in *DTNBP1* did not reach statistical significance, it should be pointed out that a haplotype comprised of rs2619539 G allele, rs875462 T allele, and rs17470454 G allele was associated with higher TD risk and a haplotype with rs760761 A, rs3213207 T, and rs16876738 C alleles with TD severity (Maes et al., 2021). Mutations of *DTNBP1* have also been associated with memory and schizophrenia through the glutamate pathway (Fallgatter et al., 2010; Hashimoto et al., 2010; Strohmaier et al., 2010).

PIP4K2A is a member of the phosphatidylinositol-5-phosphate 4-kinase family and mediates secretion, cell proliferation, differentiation, and motility (Rameh et al., 1997). It is associated with cancer (Sivakumaren et al., 2020) and it has also been examined for potential involvement in schizophrenia, but only a minor association was reported (Thiselton et al., 2010). *PIP4K2A* rs10828317, rs746203, and rs8341 have been investigated for potential association with TD in a cohort of 491 patients of Siberian origin, but only rs10828317 was associated with TD development (Fedorenko et al., 2014).

Melatonin transmembrane receptor is a G-protein, found in the brain, where it is involved in the circadian rhythm pathway (Ebisawa et al., 1999; Stauch et al., 2019). Individual polymorphisms in the genes of melatonin receptors 1A (*MTNR1A*) and 1B (*MTNR1B*) were not associated with TD. However, the haplotype comprised of *MTNR1A* rs11721818 A, rs2375801 T, and rs6553010 G alleles had a protective effect against TD (Lai et al., 2011a).

Genetic variability of *AKT1* (Zai et al., 2008; Levchenko et al., 2019), *ACE* (Segman et al., 2002b), *APOE* (Kimura et al., 2000), *PAWR* (Kim et al., 2012), *TPH* (Segman et al., 2003) and *RGS2* (Koning et al., 2012) was also examined for potential association with TD, but without statistically significant results. All of the studies are presented in this chaptercan be found in the Supplementary Table S2.

## 5 Genome-Wide Association Studies of Tardive Dyskinesia

Since 2008, seven GWAS had a goal of identifying the genetic biomarkers of TD development (Table 11). All of them were focused on patients with schizophrenia treated with antipsychotics, for whom the diagnosis and the TD were assessed according to DSM-IV and AIMS, respectively.

**TABLE 11:** Genome-wide association studies of TD.

Outcome	Number of subjects	Ethnicity	Platform	References
Association between *SLC6A11* rs4684742, *GABRB2* rs918528 and *GABRA3* rs2061051, and TD development	GWAS: 100 (50 with TD) replication cohort: 174 (36 with TD)	Japanese	Sentrix Human-1 Genotyping BeadChip (Illumina) (40,573 SNPs in 13,307 genes)	Inada et al. (2008)
Association between *HSPG2* rs2445142 and TD	GWAS: 100 (50 with TD), replication cohort: 172 (36 with TD)	Japanese	Illumina Sentrix Human-1 Genotyping 109k BeadChip	Syu et al. (2010)
Association between *GLI2* rs3943552 and TD	327 (131 with TD)	European American, African American and others	Affymetrix 500K (500,568 SNPs) and the Perlegen custom 164K chip (164,871 SNPs)	Greenbaum et al. (2010)
Association between rs7669317 located in an intergenic region and AIMS	738 (NA with TD)	European American, African American and others	Affymetrix 500K chipset (Santa Clara, CA, United States) and a Perlegen custom 164K chip	Aberg et al. (2010)
Association between SNPs in *HSPG2* and TD pathophysiology	GWAS: 100 (50 with TD), replication cohort: 172 (36 with TD)	Japanese	NA	Arinami and Inada (2011)
Significant association between *DPP6* rs6977820 and TD	122 (61 with TD), replication cohort: 174 (36 with TD)	Japanese	Illumina HumanHapCNV370 BeadChip	Tanaka et al. (2013)
Association between *GSE1* rs11639774, *TNFRSF1B* rs499646, *EPB41L2* rs6926250, and *CALCOCO1* rs4237808 and TD	1406 (280 with TD)	Han Chinese, European, African American	Illumina 1M Duo Beadchip, Affymetrix 500K “A” chipset (Nsp and Sty chips), and Perlegen’s custom 164K chip	Lim et al. (2021)

The first GWAS investigated 13,307 genes and 40,573 SNPs and was accompanied by a replication study. The cohort was comprised of 100 patients of whom 50 had TD, but no SNP was significantly associated with TD after Bonferroni correction. However, *ELOVL3* rs10748816, *BCOR* rs6609051, *TCP10L* rs7281019, *CBLC* rs10419669, *SLC38A1* rs1444590, *EHF* rs286925, *TBCD* rs3744165, *RBM17* rs2274359, *DLG5* rs1058198, *ABCC8* rs886292, *MAN1A2* rs2306444, *EDIL3* rs13153252, *ANXA13* rs4242345, *SMYD3* rs6426327 polymorphisms showed some initial potential, so authors attempted to evaluate their significance in a replication cohort, which included 174 (36 with TD) Japanese patients. *SLC6A11* rs4684742, *GABRB2* rs918528, and *GABRA3* rs2061051 were all statistically significant in both the GWAS and the replication study, indicating that the GABA receptor signaling pathway may be involved in TD pathophysiology (Inada et al., 2008). The involvement of the GABA receptor signaling pathway in TD pathophysiology was also highlighted in a canonical pathway-based analysis within a GWAS published in 2010. The GWAS included 100 Japanese schizophrenia patients (50 with treatment-resistant TD) and the findings were validated in an independent cohort of 172 patients (36 with treatment-resistant TD), identifying an association between several polymorphisms in the *HSPG2* gene and TD (Arinami and Inada 2011).

Association between TD and *HSPG2* rs2445142 was observed in an independent GWAS that studied a cohort of 100 Japanese schizophrenia patients that included 50 patients with TD. The study also included a validation cohort of 172 patients (36 with TD), who were genotyped for the following promising polymorphisms: *PLOD1* rs7529452, *HSPG2* rs2445142, *COL11A1* rs1934712, *MAN1A2* rs2306444, *TBX15* rs869807, *DUSP10* rs6668395, *SMYD3* rs6426327, *EML4* rs4558632, *ASB3* rs6714424, *LRRTM4* rs2060279, *BUB1* rs11694702, *KCNH7* rs1873201, *UBE2E3* rs11688866, *STAC* rs3749279, *TBL1XR1* rs6443468, *LOC285513* rs13115988, *C9* rs700237, *MGC33648* rs832582, *EDIL3* rs13153252, *FER* rs6594324, *FAM46A* rs915125, *CDC2L6* rs2691180, *CITED2* rs9376506, *FLJ39824* rs1832445, *ZMIZ2* rs3735478, *DPP6* rs1047053, *SULF1* rs2583086, *KCNB2* rs4738269, *STARS* rs2927111, *ANGPT1* rs3019982, *ANXA13* rs4242345, *COL15A1* rs1413299, *RBM17* rs2274359, *PCDH15* rs1932596, *DLG5* rs1058198, *ELOVL3* rs10748816, *GBF1* rs2246775, *MGMT* rs765934, *ABCC8* rs886292, *EHF* rs286925, *SPCS2* rs568758, *NEU3* rs624786, *SLC38A1* rs1444590, *KIAA1906* rs1154664, *LIG4* rs1924174, *SEC10L1* rs1189827, *ITPK1* rs11625123, *VRK1* rs10140345, *GABRG3* rs2061051, *APBA2* rs3764211, *PML* rs1036673, *DNAH9* rs3809729, *FBXW10* rs4630608, *ACACA* rs2287352, *FLJ13841* rs3744165, *DLGAP1* rs474122, *HMG20B* rs12460403, *NPHS1* rs437168, *CBLC* rs10419669, *HAS1* rs8112223, *C20orf26* rs2328500, *TCP10L* rs7281019, and *LOC91464* rs2056965 (Syu et al., 2010). Except for rs2445142 *HSPG2*, no other genetic variants reached statistical significance.

The importance of *DPP6* was pointed out in a GWAS conducted on a cohort of 122 patients with schizophrenia (61 with TD) and verified in an independent cohort of 174 patients (36 with TD). *DPP6* rs6977820, located in the first intron of *DPP6*, was statistically significantly associated with TD in both GWAS and replication study. In addition, 50-weeks administration of haloperidol was associated with high *DPP6* expression in the prefrontal, striatal, hippocampal, and ventricular midbrain regions of mice. In contrast, low gene expression was observed in the human postmortem prefrontal cortex of those carrying the risk allele. The authors concluded that the increased production of DPP6 might decrease dopamine release, which lowers dopamine sensitivity (Tanaka et al., 2013). DPP6 is a membrane protein and member of serine proteases, with an affinity for specific voltage-gated potassium channels, altering their expression. It is predominantly expressed in the brain and might be involved in neuronal plasticity and amyotrophic lateral sclerosis (Jerng et al., 2004). DPP6 protein can regulate the membrane trafficking of KV4 proteins and modify channel properties (Clark et al., 2008). Importantly, KV4 channels regulate the activity of dopaminergic neurons (Tanaka et al., 2013).

A GWAS that was performed on a cohort of 738 patients with schizophrenia within the CATIE study (Clinical Antipsychotic Trial of Intervention Effectiveness) should also be mentioned, even though it focused not solely on TD, but more broadly on movement disorders. A statistically significant association was recorded between rs7669317, AIMS scores, and probable TD. This polymorphism is located in the intergenic region of the chromosome 4q24, very close to *PPA2* (Aberg et al., 2010).

An additional two-step study was performed on a cohort of 327 patients within the CATIE cohort, 131 of whom experienced TD. Authors conducted a GWAS using the genotype data from the CATIE study, followed by a validation study with the SNPs shown to be significant in the first stage. Among the 25 SNPs investigated and genotyped by Sequenom MassArray, *GLI2* rs3943552 was significantly associated with TD (Greenbaum et al., 2010). GLI2 is a transcription factor involved in the sonic hedgehog signaling pathway, which was found to play an essential role in controlling voluntary motor movement through the differentiation of midbrain dopaminergic neurons (Abeliovich and Hammond 2007; Tolosa et al., 2020).

Lastly, the largest GWAS was published in 2021. The cohort of patients was comprised of 1406 patients (208 with TD) of Han Chinese, European and African American descent. To investigate the genetic variability of the potential underlying mechanisms and pathways of TD, they applied a robust and thorough bioinformatic analysis, including meta-analysis, functional annotation, eQTLs, transcriptome-wide fine-mapping, polygenic risk score analyses and multivariate logistic regression analyses. The study revealed the importance of three genomic loci on chromosomes 1, 6 and 12 and a novel locus on chromosome 16, highlighting the importance of *TNFRSF1B, EPB41L2, CALCOCO1* and *GSE1* genes (Lim et al., 2021). TNFRSF1B is a member of the tumor necrosis factor receptor superfamily and participates in the antiapoptotic signaling pathway. It is expressed in immune cells, highlighting the importance of immune system in TD pathogenesis (Lee and Kang 2011; Lanning et al., 2016; Lim et al., 2021). EPB41L2 is a membrane protein that was found to have a protective effect on motor activity. Published data suggest that its deficiency in mice convoy motor movement dysfunctions due to its involvement in cytoskeletal binding (Saitoh et al., 2017; Lim et al., 2021). CALCOCO1 was recently found to be involved in the autophagy of endoplasmic reticulum (Nthiga et al., 2020) and the transcriptional activation of target genes in the Wnt/CTNNB1 pathway (Mizuta et al., 2014). *GSE1* encodes a proline-rich protein with coiled-coil domains, named KIAA0182 and it is regulated by miR-489-5p. In breast cancer, *GSE1* is overexpressed, while breast cancer cells’ proliferation, migration and invasion are inhibited when silencing the gene (Chai et al., 2016). Similarly, *GSE1* is overexpressed in gastric cancer cells treated with trastuzumab, while its deletion leads to reduced trastuzumab resistance of trastuzumab-resistant gastric cancer cells (Wang et al., 2021b).

## 6 Translating Pre-clinical and Clinical Findings to Clinical Practice: Where Next?

This comprehensive review of the published findings of studies in experimental animal models and clinical studies focusing on the potential association between genetic variations and TD occurrence shows a rather significant discrepancy between genes and proteins investigated in preclinical and clinical settings.

However, we were able to identify eight genes that may be implicated in the molecular pathogenesis of TD based on the findings of the preclinical studies and which also reached statistical significance in at least one clinical study. However, the results of pharmacogenetic studies were often inconclusive or even conflicting.

Probably the most interesting entity involves the VMAT2, a transporter of the monoamine neurotransmitters from the cytosol into synaptic vesicles. The levels of VMAT2 were decreased in the experimental animals with VCM (Lévesque et al., 2017) and genetic variability of *SLC18A2* was associated with TD in two different pharmacogenetic studies (Zai et al., 2013; Lu et al., 2018). One of the most promising potential treatments for TD are the VMAT2 inhibitors, such as valbenazine and deutetrabenazine, which were approved by the FDA in 2017 (Arya et al., 2019). The detection of functionally relevant *SLC18A2* variants could allow identification of potential perturbations in VMAT2 function before the treatment initiation and would offer a window of opportunity to administer VMAT2 inhibitors along with the antipsychotic treatment to prevent TD development in the first place.

Furthermore, dopamine receptors 1 and 3 were also involved in the TD development in preclinical and at least one clinical study. It was shown that DRD1 binding is decreased in capuchin monkeys which developed TD upon haloperidol treatment (Mahmoudi et al., 2014). In addition, it was shown that the *DRD1* rs4532 CC genotype is associated with increased TD risk. *DRD1* rs4532 is a promoter polymorphism, which means that it may affect gene expression, but the exact SNP function is not known yet (Lai et al., 2011b). Moreover, DRD3 binding was increased in the monkeys with TD (Mahmoudi et al., 2014). In line with this, the *DRD3* rs6280 that increases the binding affinity of the receptor was associated with a higher chance of TD development (Steen et al., 1997; Basile et al., 1999; Segman et al., 1999, Segman et al., 2002a; Liao et al., 2001; Lerer et al., 2002; Woo et al., 2002; de Leon et al., 2005; Al Hadithy et al., 2009).

As a part of the serotonergic system, HTR2A was upregulated in the capuchin monkeys with TD, treated with haloperidol (Lévesque et al., 2017). Along with that, three *HTR2A* SNPs were associated with TD development (Segman et al., 2001; Tan et al., 2001; Hsieh et al., 2011; Pozhidaev et al., 2020).

The levels of glutamate receptors GRIN2A and GRIN2B were also shown to be increased in capuchin monkeys with TD due to haloperidol treatment (Lévesque et al., 2017). Furthermore, several *GRIN2A* SNPs (Ivanova et al., 2012b; Ivanova et al., 2016b) and one *GRIN2B* SNP (Ivanova et al., 2012b) were associated with the occurrence of TD. The functionality of the *GRIN2A* and *GRIN2B* SNP is not well known. Nevertheless, they may present a promising biomarker for identifying patients with increased risk for TD development.

A critical pathway associated with TD development is also the defense against reactive oxygen species. Animal studies showed that the activity of SOD is decreased in TD affected animals, which probably explains elevated oxidative stress in TD (Patil et al., 2012; Nade et al., 2013; Thakur et al., 2015; Wang et al., 2015; Samad and Haleem 2017; Dhingra et al., 2018; Soung et al., 2018; Tsai et al., 2019; Wang et al., 2021a). The pharmacogenetic studies of candidate genes also identified a promising genetic biomarker of TD within this pathway, namely *SOD2* rs4880 (Hori et al., 2000). This SNP decreases enzyme's activity and thus increases the risk for TD development and these observations agree with the results of the animal studies.

Lastly, CYP2D6 was shown to be decreased in the animals developing TD (Miksys et al., 2017), which agrees with the pharmacogenetic studies. The latter consistently reported that carriers of *CYP2D6* with decreased metabolizing capacity had increased odds for TD development (Koola et al., 2014; Lu et al., 2020).

All the available published information regarding the impact of human genetic variations on drug response is compiled and publicly available from the Pharmacogenomics Knowledge Base (PharmGKB) (Relling and Klein 2011; Whirl-Carrillo et al., 2012, Whirl-Carrillo et al., 2021). The database provides clinically actionable gene-drug and genotype-phenotype associations and pharmacogenomic guidelines for a variety of drugs (https://www.pharmgkb.org/). In addition, it provides information about the significance of the drug-gene association, using the term “level of evidence (LOE),” which ranges from 1 to 4. Level 1A describes the strongest variant-drug association, for which there is either a clinical pharmacogenomic recommendation or an FDA-approved drug label annotation. On the other hand, LOE 4 refers to evidence that is insufficient to support associations between the genetic variant and the drug phenotype. Regarding TD, PharmGKB has three clinical annotations listing variant-antipsychotic combinations, all of them reaching LOE 3. This level of evidence indicates a low-level association, which might be supported either by one single study or several studies that failed to validate the association. According to PharmGKB clinical annotations, patients with the *COMT* rs4680 GG genotype, *HTR2A* rs6311 TT genotype, and *DPP6* rs6977820 TT genotype, who are treated with antipsychotics, have a higher chance for TD development (https://www.pharmgkb.org/disease/PA447268/clinicalAnnotation).

Based on the above, it is evident that despite the high number of clinical and preclinical studies conducted in TD, the translation of the acquired knowledge to the everyday clinical practice is still lagging. More clinical studies with larger sample sizes are needed to validate the results and provide evidence for pharmacogenomic recommendations that can be implemented into the clinical practice.

## 7 Conclusion

Our comprehensive review showed that the preclinical and clinical studies helped to elucidate some molecular mechanisms implicated in TD development and suggested that genetic variability in these pathways may provide some promising biomarkers of TD. However, the clinical studies have failed to provide sufficient evidence for establishing pharmacogenomic recommendations that would support their implementation into psychiatric clinical practice. The studies that emerged from our literature review investigated TD in patients of diverse origins, which might explain the conflicting results. Moreover, the included cohorts’ sizes were often small, which might explain the inability to replicate the findings in some cases. Additionally, in most of the studies, risk factors for TD, like demographics, health behavior, and clinical variables, have not been considered. Finally, the studies presented in this review indicate the contribution of a single gene to the development of TD, in contrast with the fundamentals of pharmacogenomics which focuses on the contribution of multiple genes and variants. Including cohorts with bigger sizes that are very well defined in terms of dose and drug duration, smoking status, alcohol or drug use, co-medication, comorbidities, and family history of psychiatric disorders in future studies will increase our understanding of the contribution of genetic factors to the emergence of TD and will lead to selecting the ideal treatment for each patient, aiming to provide a better quality of life for patients and their caregivers. Therefore, future studies integrating different approaches, such as metabolic, neurophysiological, and genetic, are needed to elucidate the potential interactions and support the development of a personalized approach for TD management and prevention.
