# Supplementary material for: Genetic Factors Associated With Tardive Dyskinesia: From Pre-clinical Models to Clinical Studies
Source: Front Pharmacol. 2022 Jan 24;12:834129. doi: 10.3389/fphar.2021.834129 (PMC8819690; doi:10.3389/fphar.2021.834129)
Supplement: Supplementary file 1 [file Table1.docx]

**Supplementary table 1. Functional animal studies in models of tardive dyskinesia in the last ten years.**

| **Gene/protein** | **Effect in TD** | **Study model** | **Drug** | **Phenotype** | **Intervention** | **Effect of the intervention** | **Reference** |
| --- | --- | --- | --- | --- | --- | --- | --- |
| CASP3  TNFα | ↑ | Male Wistar rats | Haloperidol  Clozapine  U74500A | VCM  Enhanced oxidative stress | / | / | (Bishnoi and Boparai 2012) |
| SLC6A3  CHRNA4  CHRNB2  CHRNA6 | ↓ | Adult male Sprague-Dawley rats | Haloperidol  Nicotine | VCM | / | / | (Bordia et al. 2012) |
| / | / | Male mice from 27 inbred strains | Haloperidol | VCM and other movement phenotypes | / | / | (Crowley et al. 2012a) |
| / | / | Male Wistar rats | Fluphenazine enantate | VCM  Orofacial dyskinesia | Resveratrol | Reduced VCM, but did not modify the intensity | (Busanello et al. 2012) |
| SOD  CAT  GSH | ↓ | Male Wistar rats | Reserpine | VCM  Tongue protrusions | Roots and rhizomes of Nardostachys jatamansi and its triterpenes | Elevation in the levels of SOD, CAT, and GSH  Inhibition of lipid peroxidation | (Patil et al. 2012) |
| ***GWAS study***  *CPEB2*  *BST1*  *PIT2*  *ZIC4*  *PLSCR1*  *DRD1A* | / | Male mice from 27 inbred strains | Haloperidol | VCM and other movement phenotypes | / | / | (Crowley et al. 2012b) |
| / | / | Male Sprague–Dawley rats | Haloperidol | VCM | Deep brain stimulation  Muscimol | Decrease haloperidol induced VCMs | (Creed et al. 2012) |
| *SLC1A2* | ↓ | Wistar rats | Haloperidol decanoate | VCM | Yokukansan | VCM were ameliorated  SLC1A2 expression was increased | (Sekiguchi et al. 2012) |
| AChE activity | ↓ | Male Wistar rats | Haloperidol | Orofacial dyskinesia | Vitamin B cocktail | Prevented orofacial dyskinesia development  Increased AChE activity | (de Oliveira et al. 2013) |
| *NUR77* | ↑  (in non-dyskinetic animals treated with haloperidol) | Adult capuchin (Cebus apella) monkeys | Haloperidol | Tardive dyskinesia movements | / | / | (Mahmoudi et al. 2013) |
| CAT  SOD  GSH | ↓ | Male Wistar strain rat | Reserpine | VCM, tongue protrusions and locomotor activity | Nebivolol | Decrease in VCM and tongue protrusions  Amelioration of oxidative stress | **(Nade et al. 2013)** |
| TNFα  IL-1β  LDH | ↑ | Rats | Haloperidol | VCM, tongue protrusions, facial jerking, sniffing and grooming | PPAR-ϒ agonist pioglitazone  PPAR-α agonist fenofibrate | Reversed haloperidol-induced VCMs, facial jerking, tongue protrusion, sniffing and grooming  Decreased oxidative stress and inflammation | (Grover et al. 2013) |
| GSH  CAT | ↓ |  |  |  |  |  |  |
| / | / | Male Sprague–Dawley rats | Haloperidol | VCM | Ginkgo biloba extract  Vitamin E | Lower VCM scores | (An et al. 2013) |
| DRD3 binding  PENK | ↑ | Female capuchin (Cebus apella) monkeys | Haloperidol  Clozapine | Mild TD movements | / |  | (Mahmoudi et al. 2014) |
| DRD1 binding | ↓ |  |  |  |  |  |  |
| / | / | Adult male Wistar rats | Haloperidol | Orofacial dyskinesia | Anandamide | Decreased VCM | **(Röpke et al. 2014)** |
|  |  |  |  |  | CB1 receptor antagonist SR141716A | Blocked the effects of anandamide on VCMs |  |
| CAT activity  SOD activity  GSH levels | ↓ | Male Wistar rats | Reserpine | VCM and tongue protrusion as well as facial twitching | (-) epigallocatechin-3-gallate | Amelioration of orofacial dyskinesia  Increased CAT, GSH, SOD | (Wang et al. 2015) |
| TNFα  IL-1β | ↑ | Male Wistar rats | Haloperidol | Orofacial dyskinesia | Candesartan (AT1 antagonist)  Lisinopril (ACE inhibitor) | Improvement of the haloperidol-induced behavioral abnormalities in rats  Attenuation of oxidative damage and neuro-inflammation | (Thakur et al. 2015) |
| CAT activity  SOD activity  GSH levels | ↓ |  |  |  |  |  |  |
| *S100B* | ↑ | Male Sprague Dawley rats | Haloperidol | VCM | Gingko biloba leaf extract  Vitamin E | Decrease in S100B  Decrease in VCM  Increased antioxidative capacity | (An et al. 2016a) |
| TNFα  IL-1β  IL-6 | ↑ | Male Wistar rats | Haloperidol | Orofacial dyskinesia | Lycopene | Imorovement in behavioral, biochemical, neurochemical and neuroinflammatory markers | (Datta et al. 2016) |
| GSH | ↓ |  |  |  |  |  |  |
| CAT activity | ↓ | Male Wistar rats | Fluphenazine | Orofacial dyskinesia | Ethyl acetate fraction of H. procumbens | Amelioration of oxidative stress | (Schaffer et al. 2016) |
| IL-1β  IL-6  TNFα  IFN-γ | ↑ | Male Wistar rats | Haloperidol  Risperidone | VCM | / | / | (Peroza et al. 2016) |
| IL-10 | ↓ |  |  |  |  |  |  |
| BDNF | ↓ | Male Sprague Dawley rats | Haloperidol | VCM | Gingko biloba extract  Vitamin E | Increased BDNF  Decreased VCM | (Shi et al. 2016) |
| *BAX* | ↑ | Male Sprague Dawley rats | Haloperidol | VCM | Gingko biloba extract  Vitamin E | Reversed the increase in VCM  Decreased BAX expression  Increased BAD expression | **(An et al. 2016b)** |
| *BAD* | ↓ |  |  |  |  |  |  |
| / | / | Male Wistar rats | Haloperidol | Orofacial dyskinesia and catalepsy development | Mg supplementation | Reduced the increase of orofacial dyskinesia and catalepsy time  Reversed the reactive species generation  Decreased protein carbonyl levels | (Kronbauer et al. 2017) |
| / | / | CD-1 mice | Reserpine | VCM | Losartan (AT1R antagonist)  Istradefylline A2AR antagonist) | Decrased VCM | **(Oliveira et al. 2017)** |
| SOD activity  CAT activity  GPX activity | ↓ | Albino Wistar male rats | Haloperidol | VCM | Rice bran oil | Decreased VCM  Increased activity of the antioxidant enzymes | (Samad and Haleem 2017) |
| CYP2D6 activity | ↓ | Adult male Wistar rats | Haloperidol | VCM  Catalepsy | / | / | (Miksys et al. 2017) |
| HTR2A  GRIN2A  TH  GRIN2B | ↑ | Adult female capuchin (Cebus apella) monkeys | Haloperidol decanoate  Clozapine | Tardive dyskinesia | / | / | (Lévesque et al. 2017) |
| DAT  VMAT2 | ↓ |  |  |  |  |  |  |
| MAOB activity | ↓ | Adult male Wistar rats | Fluphenazine enantate | VCM | Resveratrol | Prevented the increase in VCMs with fluphenazine | (Busanello et al. 2017) |
| / | / | Rats | Haloperidol | Orofacial dyskinesia | L-Theanine | Prevention of the orofacial dyskinesia | (Chen et al. 2018) |
| / | / | Albino Swiss mice | Haloperidol | VCM | Gabapentin | Attennuation of VCM | (Ceretta et al. 2018) |
| TNFα  IL-6  Caspase-3 | ↑ | Male Wistar rats | Reserpine | VCM, tongue protrusion, as well as the duration of facial twitching | _L_-Theanine | Reduced VCM, tongue protrusions, facial twitching  Increased the antioxidant power  Reduced inflammation | (Soung et al. 2018) |
| GSH  SOD  CAT | ↓ |  |  |  |  |  |  |
| GSH  SOD  CAT | ↓ | Wistar male albino rats | Haloperidol | VCM, tongue protrusions, catalepsy, and locomotor activity | Hesperetin | Inhibition of VCM, tongue protrusions, and catalepsy  Reversion of haloperidol-induced increase in brain oxidative stress | (Dhingra et al. 2018) |
| *IL1B*  *TNF*  *IL6*  *NOS2*  *COX2* | ↑ | Male Swiss mice | Haloperidol | Orofacial dyskinesia | Cannabidiol (CBD) | Prevented haloperidol-induced orofacial dyskinesia and inflammatory changes  CBD effects mediated by PPARγ receptors. | (Sonego et al. 2018) |
| CAT | ↓ |  |  |  |  |  |  |
| GSH  SOD  CAT | ↓ | Male Wistar rats | Haloperidol | VCM and tongue protrusion as well as the duration of facial twitching | L-theanine | Prevention of haloperidol-induced orofacial dyskinesia  Enhancement of the antioxidant power | (Tsai et al. 2019) |
| DRD3  GRK6 | ↑ | Adult female capuchin monkeys (*Cebus apella*) | Haloperidol | Mild tardive dyskinesia | / | / | (Hernandez et al. 2019) |
| β-Arrestin2 | ↓ |  |  |  |  |  |  |
| / | / | *GNAL*^+/-^ mice and their isogenic *GNAL*^+/+^ littermates | Haloperidol | Catalepsy responses and different cellular effects | / | Enhanced behavioural abnormalities in *GNAL*^+/-^ mice if comapred to the *GNAL*^+/+^ mice. | (Khan et al. 2019) |
| / | / | Male Wistar adult rats | Haloperidol | VCM, locomotion, oxidative stress and blood glucose | Cannabidiol | Attenuation of VCM | (Kajero et al. 2020) |
| / | / | Male Swiss mice | Reserpine | VCM and locomotor activity | (+)-catechin | Catechin decreased the VCMs induced by reserpine | (Reinheimer et al. 2020) |
| GSH  SOD  CAT | ↓ | Male Wistar rats | Haloperidol | VCM and tongue protrusion | Naringin | Treatment reduced haloperidol-induced increases in the frequency of VCM and TP | (Wang et al. 2021) |
| TNFα  IL-1β  IL-6  Caspase-3 | ↑ |  |  |  |  |  |  |
| CB1 | ↑ | Male Wistar rats | Haloperidol | VCM | CB_1_ receptor antagonist (AM251)  TRPV_1_ channel blocker (SB366791) | CB_1_ receptor antagonist decreased VCM, but not TRPV_1_ channel blocker. | (Röpke et al. 2021) |
| / | / | Male Wistar rats | Haloperidol | VCM | Oral acetaminophen treatment  Injection of N-(4-hydroxyphenyl)-arachidonylamide (AM404) | Haloperidol-induced VCMs were mitigated by treatment with acetaminophen and AM404 | **(Nagaoka et al. 2021)** |
|  |  | Male C57BL/6J mice  (*TRPV1* deficient and WT) |  |  | Acetaminophen | Prevented the haloperidol-induced decrease in the number of c-Fos+preproenkephalin+ striatal neurons in wild-type mice but not in TRPV1-deficient mice. |  |
|  |  |  |  |  | AM404 | Haloperidol-induced VCMs were mitigated, but the effect was not seen in TRPV1-deficient mice. |  |

Supplementary Table 2. Pharmacogenomic studies focusing on the association between genetic variations of genes involved in the pharmacokinetics and drug-induced tardive dyskinesia.

| **Genes examined** | **Genetic variations examined** | **Drugs** | **Number of subjects** | **Ethnicity** | **Diagnosis/clinical characteristic** | **Method of clinical diagnosis** | **Method of TD assessment** | **Genotyping method** | **Results** | **Reference** |
| --- | --- | --- | --- | --- | --- | --- | --- | --- | --- | --- |
| *CYP2D6* | CYP2D6*1, CYP2D6*3, CYP2D6*4, CYP2D6*5 CYP2D6*6 and CYP2D6*7 | Neuroleptic treatment | 100 | Caucasian (South-west Scotland) | Schizophrenia | NA | AIMS | NA | A non-significant trend of association between PMs and TD severity | (Andreassen et al. 1997) |
| *CYP2D6* | CYP2D6*1, CYP2D6*3, CYP2D6*4 and CYP2D6*5 | NA | 76 | Caucasian | Schizophrenia | NA | AIMS | PCR-RFLP | No statistically significant genotypic or allele frequencies | (Armstrong et al. 1997) |
| *CYP2D6* | CYP2D6*1, CYP2D6*3, CYP2D6*4 and CYP2D6*5 | Debrisoquine | 16 | Caucasian | Schizophrenia | NA | AIMS | PCR-RFLP | No statistically significant genotypic or allele frequencies | (Arthur et al. 1995) |
| *CYP1A2* | 734C/A | NA | 85 | NA | Schizophrenia | NA | AIMS | NA | Association between CC genotype and AIMS score | (Basile et al. 2000) |
| *CYP2D6* | CYP2D6*1 to CYP2D6*15and CYP2D6*17 | Antipsychotics | 172 | Caucasian (German) | Schizophrenia or schizoaffective psychosis | ICD-10 | AIMS | PCR-RFLP | No statistically significant genotypic or allele frequencies | (Brockmöller et al. 2002) |
| *CYP1A2* | 734C/A | NA | 103 | Chinese | Schizophrenia | DSM-IV | AIMS & Schooler  and  Kane  criteria | NA | No statistically significant genotypic or allele frequencies | (Chong et al. 2003b) |
| *CYP2D6, CYP3A5* | CYP2D6*1, *2, *3, *4, *5, *6, *7, *8, *9, *10, *11, *14, *15, *17, *18, *19, *20, *25, *26, *29, *30, *31, *35, *36, *37, *40, *41, *43, *45, CYP2D6 duplications, CYP3A5*3, *6 | risperidone, olanzapine,  FGAs, and quetiapine | 516 (162 with TD) | Caucasian | Schizophrenia, schizoaffective  disorder, bipolar  disorder and major depressive disorder | NA | AIMS | Allele specific PCR or Amplichip P450 microarray and others | No association between CYP2D6 and CYP3A5 alleles and TD | (de Leon et al. 2005) |
| MDR1 | rs1045642 | risperidone, olanzapine,  FGAs, and  quetiapine | 516 (162 with TD) | Caucasian | Schizophrenia, schizoaffective  disorder, bipolar  disorder and major depressive disorder | NA | AIMS | Allele specific PCR or Amplichip P450 microarray and others | No association between rs1045642 and TD severity and development | (de Leon et al. 2005) |
| MDR1 | rs1045642, rs1922242 | long-term antipsychotic treatment | 187 | Caucasian | schizophrenia | NA | AIMS | TaqMan assay | No statistically significant genotypic or allele frequencies. However, the T–A and T-T haplotypes of rs1045642 and rs1922242 were associated with lower and higher AIMS scores, respectively | (De Luca et al. 2009) |
| *CYP2D6* | CYP2D6*1, CYP2D6*3 and CYP2D6*4 | Antipsychotics | 37 | Americans | Schizophrenia | DSM-IV | AIMS | Allele specific PCR | Most smokers with CYP2D6*1/*3, *4 genotype had TD | (Ellingrod et al. 2002) |
| *CYP2D6, CYP1A2* | CYP2D6*10, CYP1A2*1F | FGAs | 182 (91 with TD) | Chinese | Schizophrenia | DSM-IV | AIMS | PCR-RFLP (HphI for CYP2D6*10 and Bsp120I for CYP1A2*1F) | Association between the T allele of CYP2D6*10 and C allele of CYP1A2 and TD | (Fu et al. 2006) |
| *CYP2D6, CYP1A2* | CYP2D6*1, *2, *3, *4,  *5, *6, *9, *10, *17, *29, *41, CYP1A2*1F | olanzapine, quetiapine, risperidone, ziprasidone and perphenazine | 750 (180 with TD) | Caucasian, African American | Schizophrenia | NA | Schooler-Kane criteria | TaqMan | No association between CYP2D6 and CYP1A2 and TD | (Grossman et al. 2008) |
| *CYP2D6* | CYP2D6*2,  CYP2D6*3, CYP2D6*4, CYP2D6*10, and CYP2D6*12 | Neuroleptics | 309 (41 with TD) and 196 controls | Japanese | Schizophrenia | DSM-III-R | Japanese version of AIMS | PCR-RFLP | No association between CYP2D6 alleles and TD | (Inada et al. 2003) |
| *CYP17* | rs743572 | NA | 146 | Caucasian (Siberian) | schizophrenia or schizotypal disorder | ICD-10 | AIMS | TaqMan assay | Association between rs743572 CC genotype and lower risk for TD | (Ivanova et al. 2014) |
| *CYP1A2* | rs762551 (CYP1A2*1F) | FGAs and SGAs | 319 and 117 healthy individuals | Caucasian (Siberian) | Schizophrenia | NA | AIMS | Real-Time PCR | Association between rs762551 and TDlt; smoking may decrease the plasma levels of antipsychotics, through increased CYP1A2 metabolism | (Ivanova et al. 2015) |
| *CYP2D6,*  *CYP1A2* | CYP2D6*3,  CYP2D6*4,  CYP1A2*1F | monotherapy or combination of haloperidol, chloroprotixene, chloropromazin, trifluoperazin, zuclopenthixol, risperidone | 353 (140 with TD) | Caucasian | Schizophrenia | IDC-10 | AIMS | real time PCR with fluorescent probes | Association between CYP2D6*4 and limbotruncal TD, association between  CYP1A2*1F and TD development | (Ivanova et al. 2016a) |
| *CYP2D6* | CYP2D6*1, CYP2D6*3 and CYP2D6*4 | Zuclopenthixol | 52 | Estonian or Russian | Schizophrenia or schizoaffective  disorder | ICD-10 | AIMS | Allele  specific PCR | A trend of association between patients carrying at least one mutated CYP2D6 and increased TD risk | (Jaanson et al. 2002) |
| *CYP2D6* | CYP2D6*3, CYP2D6*4 and CYP2D6*5 | Neuroleptic treatment | 26 | Caucasian (Austrian origin) | Schizophrenia | DSM IV | Schooler-Kane criteria | NA | Association between heterozygosis and higher TD incidence | (Kapitany et al. 1998) |
| *CYP2D6* | CYP2D6*41,  CYP2D6*3,  CYP2D6*4,  CYP2D6*5, CYP2D6*2 | FGAs | 13 with TD and 57 without TD | Caucasian (European) | Schizophrenia | DSM-IIIR | AIMS & Schooler  and  Kane  criteria | PCR-RFLP | TD risk is associated with an increase in the number of functional CYP2D6 genes | (Koola et al. 2014) |
| *CYP2D6* | CYP2D6*1 and CYP2D6*10 | NA | 76 | Chinese | schizophrenia | DSM-IV | AIMS & Schooler  and  Kane  criteria | PCR-RFLP | No statistically significant association between CYP2D6*10 genotypes and TD in men. However, a significant increase in CYP2D6*10 allele frequency in female patients with TD. | (Lam et al. 2001) |
| *CYP2D6* | CYP2D6*10 | NA | 216 (113 with TD) | Chinese | Schizophrenia | NA | AIMS | NA | Modest association between CYP2D6*10 genotypes and TD | (Liou et al. 2004) |
| *CYP2D6* | CYP2D6*1, CYP2D6*3, CYP2D6*4, CYP2D6*5 and CYP2D6*6 | Antipsychotic drugs | 109 | Caucasian (German) | Schizophrenia or schizoaffective disorder | DSM IV | TDRS | SSCP analysis | No statistically significant genotypic or allele frequencies | (Lohmann et al. 2003) |
| *CYP2D6* | CAUS: rs1135840, rs16947, rs28371706, rs28371725, rs35742686,  rs3892097, rs5030655, rs5030656 and rs1065852 (*CYP2D6*) | FGAs, SGAs, or no exposure | CAUS: 148 (63 with TD),  PGx: 58 (19 with TD) | Caucasian (European) | Schizophrenia or schizoaffective disorder | DSM-III-R or DSM-IV | AIMS or the modified HSDS | CAUS: TaqMan assay (SNPs), Real-Time PCR (copy number of *CYP2D6*),  PGx: multiplex Luminex xTAG bead array | *CYP2D6* UMs and PMs have higher risk for TD development and severity in comparison with IMs and EMs | (Lu et al. 2020) |
| *CYP1A2* | 734C/A and -2964G/A | NA | 199 | Japanese | Schizophrenia | NA | AIMS | PCR-RFLP | No statistically significant genotypic or allele frequencies | (Matsumoto et al. 2004a) |
| *CYP2D6* | CYP2D6*1 to CYP2D6*1, CYP2D6*10B, CYP2D6*14, CYP2D6*18, CYP2D6*19, CYP2D6*25, CYP2D6*26, CYP2D6*31, CYP2D6*36 and CYP2D6*41 | Conventional neuroleptics such as haloperidol, chlorpromazine, thioridazine, trifluperazine, fluphenazine, perphenazine, zuclopenthixol and pimozide | 202 | Korean | Schizophrenia | DSM-IV | AIMS | Affymetrix CYP450  GeneChip  system | Males with at least  one decreased or loss of function allele have a moderately greater chance of developing TD than males with only wild-type alleles | (Nikoloff et al. 2002) |
| *CYP2D6* | CYP2D6*3, CYP2D6*4 and CYP2D6*10 | Haloperidol, levomepromazine, bromperidol, mosapramine, chlorpromazine, clocapramine | 100 | Japanese | Schizophrenia | DSM-IV | AIMS | PCR-RFLP | Statistically significant difference of CYP2D6*10 alleles and patients with and without TD. Significant genotypic and allelic associations with dichotomized total AIMS scores. Significant association between CYP2D6*10 genotype and total AIMS score, and modest association with TD occurrence | (Ohmori et al. 1998) |
| *CYP2D6* | CYP2D6*2 | NA | 99 | Japanese | Schizophrenia | NA | NA | NA | No statistically significant genotypic or allele frequencies | (Ohmori et al. 1999) |
| *CYP2D6* | CYP2D6*1, *2, *3, *4, *5, *6, *8, *9, *10, *11, *12, *14, *15, and CYP2D6 duplications | haloperidol, fluphenazine, zuclopenthixol or risperidone | 131 (23 with TD) | Caucasian (Slovenian) | schizophrenia or schizoaffective disorder alleles. | DSM IV, PANSS | AIMS | long-PCR (CYP2D6 gene duplications and deletion), nested PCR-RFLP low resolution (CYP2D6*3 and *4) high resolution (CYP2D6*2, *6, *8, *9, *10, *11, *12, *14 and *15), allele-specific PCR | No association between CYP2D6 alleles and TD | (Plesnicar et al. 2006) |
| *CYP1A2* | 734C/A | NA | 119 | Caucasian (German) | Schizophrenia | NA | AIMS | NA | No statistically significant genotypic or allele frequencies | (Schulze et al. 2001) |
| *CYP2D6* | CYP2D6*1, *3, *4, *5, *6, duplications in CYP2D6 | haloperidol, perphenazine, levomepromazine, fluphenazine, chlorpromazine, thioridazine, and zuclopenthixol | 71 (15 with TD) | Caucasian (European) | Paranoid, disorganized, undifferentiated, catatonic, residual schizophrenia | DSM IV | AIMS | allele-specific PCR, PCR-RFLP (XbaI), long PCR | No association between CYP2D6 alleles and TD | (Scordo et al. 2000) |
| *CYP17* | T/C in the 5’ promoter region | FGAs | 113 (55 with TD) | Ashkenazi and non-Ashkenazi | Schizophrenia | DSM-IV | AIMS | PCR-RFLP (MspAI) | Association between the carriers of CYP17 A2-A2 genotype and risk of abnormal orofacial and distal involuntary movements | (Segman et al. 2002a) |
| *CYP1A2* | CYP1A2*2, *4, *5, *6, CYP1A2*1C, CYP1A2*1F | FGAs and SGAs | 335 (96 with TD) and 250 controls | North India | Schizophrenia | DSM IV | AIMS | SnaPshot method (CYP1A2*2, *4, *5, *6), PCR-RFLP (CYP1A2*1C and CYP1A2*1F) | Carriers of CYP1A2*1C (G4A) variant allele who had received only FGAs and were smokers had increased severity of TD | (Tiwari et al. 2005a) |
| *CYP3A4, CYP2D6* | CYP3A4*1B and CYP2D6*4 | FGAs and SGAs | 335 (96 with TD) | North India | Schizophrenia | DSM IV | AIMS | melting curve analysis (CYP3A4*1B), PCR-RFLP (CYP2D6*4) | No association between CYP3A4*1B and CYP2D6*4 and TD | (Tiwari et al. 2005b) |

*Hillside Simpson Dyskinesia Scale (HSDS), Abnormal Involuntary Movement Scale (AIMS), High resolution melting analysis (HRM), Research Diagnostic Criteria for Tardive Dyskinesia (RDC-TD), limb-truncal dyskinesia (TDlt),

Please note that the studies are presented alphabetically, by the first author’s name.

Supplementary table 3. Pharmacogenomic studies focusing on the association between genetic variations of genes involved in pharmacodynamics and drug-induced tardive dystonia.

| **Genes examined** | **Genetic variations examined** | **Number of subjects** | **Ethnicity** | **Diagnosis/**  **clinical characteristic** | **Method of clinical diagnosis** | **Method of TD assessment** | **Drugs** | **Genotyping method** | **Results** | **Reference** |
| --- | --- | --- | --- | --- | --- | --- | --- | --- | --- | --- |
| *DRD3, HTR2A, HTR2C* | rs6280 (*DRD3*), rs6311 (*HTR2A*), rs6318 (*HTR2C*) | 146 | Caucasian (Russian) | Schizophrenia or schizotypal disorder | ICD‐10 | AIMS | NA | TaqMan assay | association between rs6318 and rs6280 with TDlt | (Al Hadithy et al. 2009) |
| *NA* | NA | WES: 82 (39 with TD), replication: 140 (61 with TD) | WES: Jewish Israeli (Ashkenazi or non-Ashkenazi), replication: European,  African | WES: Schizophrenia, replication: schizophrenia or schizoaffective disorder | WES: DSM-IV-TR, replication: DSM-III-R or  DSM-IV | AIMS | FGAs | WES | Association between rs567070433 in *RIMS2* gene and TD | (Alkelai et al. 2019) |
| *DRD3, DRD4, GRIN2B,*  *GRIN2A,*  *HSPG2,*  *HTR2C and*  *NQO1* | rs7313149, rs2192970, rs2300242, rs10845838, rs12300851, rs220599, rs10772715, rs12827536 (*GRIN2B*),  rs11866328, rs11646587, rs7196095, rs8049651, rs9989388, rs9921541, rs4782039, rs7190619, rs9788936, rs8057394, rs11644461, rs7192557, rs1345423, rs1650420 (*GRIN2A*),  rs2270697, rs2445142, rs6698486 (*HSPG2*), rs9817063, rs2134655, rs963468, rs324035, rs3773678, rs167771, rs11721264, rs167770, rs7633291, rs1800828 (*DRD3*), rs3758653 (*DRD4*), rs569959, rs17326429, rs12858300, rs4911871, rs5946189, rs1801412 (*HTR2C*), rs1800566 (*NQO1*) | 168 | Caucasian (European) | schizophrenia | DSM-IV, CGI-SCH SI | AIMS & Schooler and Kane’s criteria | NA | Veracode and TaqMan Assays | Nominal significance for rs1345423, rs7192557, rs1650420 and rs11644461 with TD, rs7192557, rs1650420 and rs4911871 with orofacial dyskinesia, rs1345423, rs7192557, rs1650420, and rs11866328 with limb truncal dyskinesia. However, no statistically significant associations after controlling for multiple testing | (Bakker et al. 2012) |
| *DRD3* | rs6280 | 112 | Caucasian, Asian, and African American | Schizophrenia | DSM-IIIR | AIMS & Schooler-Kane criteria & modified HSDS | FGAs | PCR-RFLP (MscI) | Statistically significant association between glycine variant and TD | (Basile et al. 1999) |
| *HTR2A* | rs6313, rs6311,  and rs6314 | 136 | Caucasian, African American | Schizophrenia | DSM-IIIR | AIMS | FGAs | PCR-RFLP (Mspl, Bsml) | No statistically significant genotypic or allele frequencies | (Basile et al. 2001) |
| *CHRM1, CHRM2* | rs2075748, rs544978, rs2067477, rs2067479, rs2186410, rs542269 (*CHRM1*), rs2061174, rs324650, rs2350780, rs1824024, rs7810473, rs2350786, rs324640, rs1378650 (*CHRM2*) | 472 | Caucasian (Russian) | Schizophrenia | ICD‐10 | AIMS | FGAs and SGAs | Real time PCR | rs2061174 and rs1824024 frequencies were lower in TD patients, indicating protective effect for TD development | (Boiko et al. 2020) |
| *HTR2A*, *CYP1A2* | rs6311(*HTR2A*) and rs762551 (*CYP1A2*) | 127 (47 with TD) and 100 healthy controls | Caucasian (Turkish) | Schizophrenia | DSM-IV | AIMS | anti-  psychotic and anticholinergic medication | PCR-RFLP (MspI and Bsp1201) | No association between CYP1A2 polymorphism and TD. However, cumulative exposure to antipsychotics for every year and rs6311 AA genotype of HTR2A gene are risk factors for TD. | (Boke et al. 2007) |
| *TNFA, SOD2, GPX1, CAT* | rs1800629 (*TNFA*), rs4880 (*SOD2*), rs1050450 (*GPX1*), rs1001179 and rs10836235 (*CAT*) | 52 (6 with TD) | Caucasian (European) | Schizophrenia | DSM-IV, PANSS, GAF | AIMS | haloperidol | TaqMan assay | No statistically significant genotypic or allele frequencies | (Bošković et al. 2013) |
| *DRD2* | rs1800497 | 93 | Chinese (Taiwanese) | Schizophrenia | DSM-III-R | AIMS & Schooler-Kane criteria | pro-  longed  neuroleptics  treatment | PCR-RFLP | Significant genotypic and allele differences between female patients with and without TD | (Chen et al. 1997) |
| *5-HTT (SLC6A4)* | 5-HTTLPR VNTR | 188 | Chinese | Schizophrenia | NA | AIMS | Neuroleptics | NA | No statistically significant genotypic or allele frequencies | (Chong et al. 2000) |
| *DRD2 & DRD3* | rs801028 (*DRD2*), rs6280 (*DRD3*) | 117 | Chinese | Schizophrenia | DSM-IV | AIMS | NA | NA | No statistically significant genotypic or allele frequencies for none of the studied polymorphisms | (Chong et al. 2003a) |
| *DRD2, DRD3, GSTT1, GSTM1* | rs1801028 and  rs1799732 (DRD2), rs6280 (DRD3), GSTM1 and GSTT1 deletions | risperidone, olanzapine,  FGAs, and  quetiapine | 516 (162 with TD) | Caucasian | Schizophrenia, schizoaffective  disorder, bipolar  disorder and major depressive disorder | NA | AIMS | Allele specific PCR or Amplichip P450 microarray and others | Association between rs6280 and TD severity, and GSTM1 absence and TD development | (de Leon et al. 2005) |
| *PIP5K2A* | rs10828317, rs746203, rs8341 | 491 (131 with TD) | Caucasian (Siberian) | Schizophrenia or schizotypal disorder | ICD-10 | AIMS | NA | TaqMan assay | A significant association between rs10828317 and TD | (Fedorenko et al. 2014) |
| *DRD3* | rs6280 | 84 | Caucasian, Afro-caribbean, Middle  Eastern,  and Vietnamese | Schizophrenia | PSE  and  High  Royds  Evaluation of  Negativity | Modified  Rogers  Scale | 49 under clozapine | PCR-RFLP (MscI) | No statistically significant genotypic or allele frequencies | (Gaitonde et al. 1996) |
| *DRD3* | rs6280 | 65 | Chinese | Schizophrenia | DMS-IV, BPRS | AIMS & Schooler and Kane’s criteria | chlorpromazine equivalent | PCR-RFPL (MscI) | No statistically significant genotypic or allele frequencies | (Garcia-Barceló et al. 2001) |
| *HSPG2* | rs2445142, rs2124368, rs4738269, rs886292 and rs2061051 | Jewish Israeli: 166, CATIE: 327 | Jewish Israeli, European or Hispanic, African Americans and mixed | Schizophrenia | DSM-IV | RDC-TD, AIMS | NA | Sequenom Mass array | Association between rs2445142 G allele of *HSPG2* and TD risk in Jewish Israeli and CATIE subjects | (Greenbaum et al. 2012) |
| *HTR2A, COMT, 5-HTT (SLC6A4)* | rs6313, rs6311 (*HTR2A*), high/low activity (*COMT*), 5-HTTLPR VNTR (*5-HTT*) | 32 | Caucasian (Turkish) | Schizophrenia | DSM-IV | ESRS | NA | NA | No statistically significant genotypic or allele frequencies | (Herken et al. 2003) |
| *MnSOD* | rs4880 | 286 (30 with TD) and 243 controls | South African Xhosa | Schizophrenia | DSM-IV | AIMS | FGAs and SGAs | HEX-SSCP analysis | Genotypic association with TD and AIMS scores | (Hitzeroth et al. 2007) |
| *MnSOD* | rs4880 | 192 (39 with TD) and 141 controls | Japanese | Schizophrenia | DSM-IV | AIMS | Haloperidol, levomepromazine, bromperidol, chlorpromazine, zotepine, propericia-  Zine, Sulpiride, mosapramine and combination | PCR-RFLP (BsaWI) | Significant difference  in genotypic distribution between patients with and without TD. Association between decreased rs4880 9Ala allele frequencies and TD | (Hori et al. 2000) |
| *DRD2* | rs1801028, rs1799732, rs1800497 | 44 | NA | Schizophrenia | NA | AIMS | NA | NA | No statistically significant genotypic or allele frequencies | (Hori et al. 2001) |
| *HTR2A, HTR2C, SLC6A4* | rs6313 (*HTR2A*), rs6318 (*HTR2C*), HTTLPR (*SLC6A4*) | 32 | Han Chinese (Taiwanese) | chronic schizophrenia, | NA | AIMS | FGAs | NA | Association between the T allele of rs6313 and TD development | (Hsieh et al. 2011) |
| *DBH* | rs72393728 (5’-Ins/Del in *DBH*) | 741 (345 with TD) | Han Chinese | Schizophrenia | DSM-IV | AIMS, Research Diagnostic Criteria in DSM-IV, in combination with the criteria of Schooler and Kane | clozapine, risperidone, perphenazine, sulpiride, chlorpromazine, haloperidol, and others | agarose gel electrophoresis | No statistically significant genotypic or allele frequencies | (Hui et al. 2015) |
| *DBH* | rs72393728 (5’-Ins/Del in *DBH*) | 741 (345 with TD) | Han Chinese | Schizophrenia | DSM-IV | AIMS | clozapine, risperidone, perphenazine, sulpiride, chlorpromazine, haloperidol | agarose gel electrophoresis | No statistically significant genotypic or allele frequencies | (Hui et al. 2017) |
| *ADORA2A* | 2592C/Tins | 146 | Caucasian (Siberian) | Schizophrenia or schizotypal disorder | ICD-10 | AIMS | NA | TaqMan assay | No statistically significant genotypic or allele frequencies | (Ivanova et al. 2012a) |
| *GRIN2A, GRIN2B, DRD3, DRD4, HTR2C, HSPG2, MnSOD, NQO1* | rs9989388, rs7190619, rs8049651, rs7196095, rs9921541, rs7192557, rs9788936, rs7206256, rs11866328, rs4782039, rs1345423, rs11644461, rs11646587, rs8057394, rs1650420 (*GRIN2A*), rs12300851, rs220599, rs7313149, rs12827536, rs10772715, rs10845838, rs1805481, rs2192970, rs2300242 (*GRIN2B*), rs167770, rs167771, rs324035, rs963468, rs1800828, rs2134655, rs3773678, rs7633291, rs9817063, rs11721264 (*DRD3*), rs3758563 (*DRD4*), rs6318c, rs569959, rs1801412, rs3813929, rs4911871 (*HTR2C*), rs2270697 (*HSPG2*), rs4880 (*MnSOD*), rs1800566 (*NQO1*) | 431 | Caucasian (European) | schizophrenia or schizotypical disorders | ICD-10 | AIMS | NA | Veracode Assay | rs11646587, rs7206256 and  rs1345423 were associated with orofacial TD, rs7190619 and rs9788936 were associated with TDlt, rs2192970 and  rs1345423 were associated with overall TD | (Ivanova et al. 2012b) |
| *GRIN2A, GRIN2B, DRD3, HTR2C, DRD4* | 43 tag SNPs | Cohort 1: 431  Cohort 2: 168 | Caucasian (Siberia and Dutch) | Cohort 1: schizophrenia or schizotypal disorder,  Cohort 2:  Axis I, schizophrenia, psychosis, affective disorders,  another Axis I diagnosis, and no Axis I diagnosis | Cohort 1:  ICD-10, Cohort 2: DSM-IV | AIMS | NA | Veracode Assay | Statistically significant associations between rs1345423 in *GRIN2A* and all TD types, including TDlt, in both cohorts | (Ivanova et al. 2016b) |
| *DRD2* | rs1799978, rs1799732, rs1079597, rs1800498, rs1801028, rs1800497, Val96Ala, rs1800499, rs1800496 | 665 | Caucasian (German) | schizoparanoid, schizoaffective, residual, catatonic, disorganized, schizophrenic, undifferentiated and unclassified | DSM-IV | AIMS | FGAs | Sequencing | No statistically significant genotypic or allele frequencies | (Kaiser et al. 2002) |
| *HTR3A* | rs1062613 | 280 (105 with TD) | Korean | Schizophrenia | DSM | AIMS | FGAs and SGAs | HRM | No statistically significant genotypic or allele frequencies | (Kang et al. 2013) |
| *PAWR* | rs7979987, rs4842318, rs17005769 | 280 (105 with TD) | Korean | Schizophrenia | DSM-IV | RDC-TD | FGAs and SGAs | HRM | No statistically significant genotypic or allele frequencies | (Kim et al. 2012) |
| *ApoE* | ApoE ε2, ε3, and ε4 | 333 (62 with TD) and 191 controls | Japanese | Schizophrenia | DSM IV | NA | NA | polyacrylamide gel isoelectricfocusing | No statistically significant genotypic or allele frequencies | (Kimura et al. 2000) |
| *DRD2, DRD3, 5HTR2A, 5HTR2C, COMT, NQO1, GSTP1, RGS2 and MnSOD* | rs1800497, rs6277, rs1799732, and rs1800498 (*DRD2*), rs6280 (*DRD3*), rs6313 and rs6314 (*5HTR2A*), rs6318 and rs3813929 (*5HTR2C*), rs4680 (*COMT*), rs1800566 (*NQO1*), rs1695 (*GSTP1*), rs4606 (*RGS2*), and rs4880 (*MnSOD*) | 402 (188 with TD) | Caucasian (Northern European) | Schizophrenia, Schizoaffective disorder, Schizophreniphorm disorder, Delusional disorder,  Psychotic disorders NOS, others | DSM-IV | AIMS | FGAs and SGAs | Sequencing | No statistically significant genotypic or allele frequencies | (Koning et al. 2012) |
| *ESR 1* | NA | 246 (118 with TD) | Han Chinese | Schizophrenia | DSM-IV | AIMS | FGAs, SGAs | PCR-RFLP (Pvu II and Xba I) | A trend of association between the genotypes determined by Pvu II enzyme and TD | (Lai et al. 2002) |
| *MRNR1A, MTNR1B* | 6 Tag SNPs  rs11721818, rs2375801, rs6553010 (*MTNR1A*), rs4753426,  rs10830963, rs3781637 (*MTNR1B*) | 418 (256 with TD) | Han Chinese | Schizophrenia | DSM-IV | AIMS | FGAs | TaqMan assay | rs11721818A, rs2375801T, and rs6553010G haplotype has a protective effect | (Lai et al. 2011a) |
| *DRD1* | rs5326, rs4532 and rs265975 | 220 | Han Chinese | Schizophrenia | DSM-IV | AIMS & Schooler and Kane’s criteria | long-term antipsychotic treatment | TaqMan assay | Significant association between GG genotype of rs4532 and TD | (Lai et al. 2011b) |
| *NRXN1* | rs17041112, rs10490162, rs1400882, rs12467557, and rs1045881 | 178 (72 with TD) | Caucasian (European) | schizophrenia or schizoaffective disorder | DSM‐IIIR/IV | AIMS | FGAs | custom‐designed Illumina platform | No statistically significant genotypic or allele frequencies | (Lanning et al. 2017) |
| *DRD3* | rs6280 | 780 patients (317 with TD) | Caucasian (Northern Italian, German, Austrian), Israeli, African  American | Schizophrenia or schizoaffective  disorder | DSM-IV, ICD-9 | Research Diagnostic Criteria for TD (Schooler and Kane 1982), AIMS, Tardive Dyskinesia Rating Scale | NA | PCR-RFLP (Mscl) | Association between Gly allele and TD | (Lerer et al. 2002) |
| *AKT1, GSK3B* | rs334558 (*GSK3B*), rs3730358 and rs1130214 (*AKT1*) | 449 (121 with TD) | Caucasian (Siberian) | Schizophrenia | ICD‐10 | AIMS | NA | TaqMan | No statistically significant genotypic or allele frequencies | (Levchenko et al. 2019) |
| *DRD3* | rs6280 | 115 | Chinese (Taiwanese) | Schizophrenia or schizoaffective disorder | DSM-IV | AIMS | FGAs | PCR-RFPL (MscI) | Significant association between rs6280 heterozygotes and TD | (Liao et al. 2001) |
| *COMT, MAOA* | rs4680 (*COMT*), 30-bp repeat in the  promoter region of *MAOA* | 206 (97 with TD) | Han Chinese | Schizophrenia | DSM-IV | AIMS | NA | NA | No statistically significant genotypic or allele frequencies | (Li et al. 2013) |
| *DRD2* | rs1799732, rs1079597, rs1800498, rs1801028 and rs1800497 | 253 (126 with TD) | NA | schizophrenia | NA | NA | FGAs | NA | Association between rs1800497, rs1079597 and their haplotypes with TD. B2B2 genotype, B2 allele,  A2A2 genotype and A2 allele had increased risk for  TD. | (Liou et al. 2006) |
| *DRD3* | rs6280 | 75 (32 with TD) | Caucasian (European) | Schizophrenia | DSM-III-R | AIMS | FGAs | PCR-RFPL (MscI) | No statistically significant trend of association between rs6280 Gly-allele and TD | (Løvlie et al. 2000) |
| *DISC1, DRD2, SLC18A2* | rs2492367, rs3738398, rs1322784, rs11122359, rs821597, rs701158, rs3738401, rs6675281, rs821616 (*DISC1*), rs6277, rs1800497 (*DRD2*), rs363224 (*SLC18A2*) | 174 (74 with TD) | Caucasian (European) | Schizophrenia or schizoaffective disorder | DSM-III-R or DSM-IV | AIMS, HSDS | FGAs | TaqMan assay | Interaction between rs363224 (*SLC18A2*) and rs11122359 (*DISC1*) genotypes with TD severity | (Lu et al. 2018) |
| *DTNBP1* | rs760761, rs3213207, rs16876738, rs2619539, rs875462, rs17470454 | 136 (59 with TD) | Caucasian (European) | schizophrenia or schizoaffective disorder | DSM-III-R or DSM-IV | AIMS or the modified HSDS | FGAs, SGAs, or no exposure | TaqMan assay | Association between the haplotype rs2619539G, rs875462T, and rs17470454G with higher TD risk for TD and the haplotype rs760761A,  rs3213207T, and rs16876738C with TD severity | (Maes et al. 2021) |
| *COMT, MAOA and MAOB* | rs4680 (*COMT*), 30-bp repeat  in the promoter of MAOA, rs1799836 (*MAOB*) | 206 | Japanese | Schizophrenia | DSM-IV | AIMS & Schooler-Kane criteria | FGAs | PCR-RFLP (*Mae*III) | No statistically significant genotypic or allele frequencies | (Matsumoto et al. 2004b) |
| *OPRM1, OPRD1* | rs1799971 (*OPRM1*), 921T/C (*OPRD1*) | 216 | Japanese | Schizophrenia | DSM-IV | AIMS | NA | PCR-RFLP (MaeIII) | Association between lower frequency of rs1799971 G allele and TD | (Ohmori et al. 2001) |
| *HTR6* | rs1805054 | 173 | Japanese | Schizophrenia | NA | AIMS | NA | PCR-RFLP | No statistically significant genotypic or allele frequencies | (Ohmori et al. 2002) |
| *DRD2* | rs1799732, rs1800497, rs1800498,  rs1801028, rs6275 | 100 | Korean | Schizophrenia | Korean version of DSM | AIMS & Schooler and Kane’s criteria | FGAs | NA | No statistically significant genotypic or allele frequencies | (Park et al. 2011) |
| *HTR1A,*  *HTR1B, HTR2A, HTR2C,*  *HTR3A, HTR3B, HTR6* | rs6295, rs1364043, rs10042486, rs1800042, rs749099 (*HTR1A*), rs6298, rs6296, rs130058 (*HTR1B*),  rs6311, rs6313,  rs6314, rs7997012, rs1928040, rs9316233, rs2224721 (*HTR2A*),  (rs6318, rs5946189, rs569959, rs17326429, rs4911871, rs3813929,  rs1801412, rs12858300 (*HTR2C*), rs1062613, rs33940208, rs1176713 (*HTR3A*), rs1176744 (*HTR3B*) and rs1805054 (*HTR6*) | 449 (121 with TD) | Caucasian | Schizophrenia | ICD-10 | AIMS | NA | SEQUENOM Mass Array (combination of spectrometry with end-point PCR) | Significant associations between rs1928040 (*HTR2A*) and rs1801412 (*HTR2C*) in female patients with the orofacial type of TD | (Pozhidaev et al. 2020) |
| *DRD3* | rs6280 | 157 | Caucasian (German) | schizophrenia or schizoaffective disorder | NA | TDRS | long-term neuroleptic medication | NA | No statistically significant genotypic or allele frequencies | (Rietschel et al. 2000) |
| *DRD3* | rs6280 | 100 | Caucasian | Schizophrenia | NA | AIMS | long-term anti-psychotic drug therapy | NA | Association between rs6280 Gly-allele and TD | (Steen et al. 1997) |
| *DRD3* | rs6280 | 53 | Ashkenazi and non-Ashkenazi | Schizophrenia | NA | AIMS | Antipsychotics | NA | Association between rs6280 Ser/Gly genotypes and TD | (Segman et al. 1999) |
| *HTR2C, DRD3* | rs6318 (*HTR2C*), rs6280 (*DRD3*) | 55 | NA | Schizophrenia | NA | NA | FGAs | NA | Association between rs6318 Ser allele and TD | (Segman et al. 2000) |
| *HTR2A* | rs6313, rs6311,  and rs6314 | 121 (59 with TD) and 96 controls | Jewish (Ashkenazi or non-  Ashkenazi) | Schizophrenia | DSM-IV | RDC-TD | FGAs | PCR-RFLP | Association between rs6313 C and rs6311 G alleles, and CC and GG genotypes respectively, with TD | (Segman et al. 2001) |
| *ACE* | insertion/deletion in the 16th intron | 113 (55 with TD) and 87 controls | Jewish Israeli (Ashkenazi or non-Ashkenazi) | Schizophrenia, major depression, and bipolar disorder | DSM-IV | AIMS | NA | NA | No statistically significant genotypic or allele frequencies | (Segman et al. 2002b) |
| *DRD3* | rs6280 | FGAs | 113 (55 with TD) | Ashkenazi and non-Ashkenazi | Schizophrenia | DSM-IV | AIMS | PCR-RFLP (MspAI) | Association between the carriers of DRD3Gly allele and risk of abnormal orofacial and distal involuntary movements | (Segman et al. 2002a) |
| *DRD2, DRD4, HTR6, SLC6A3, SLC6A4, TPH* | rs1800497, rs1799732, rs1801028 (DRD2), 40 bp VNTR, a G2319A  transversion (SLC6A3), 120 bp repeat located 1.2 kb upstream  of the initiation codon, 48 bp VNTR in exon 3 (DRD4), - rs1805054, (HTR6), 5-HTTLPR and rs1800532 (TPH) | FGAs | 122 (59 with TD) | Ashkenazi and non-Ashkenazi | schizophrenia | DSM-IV | AIMS | PCR-RFLP | No statistically significant genotypic or allele frequencies | (Segman et al. 2003) |
| *NOS1* | C/T polymorphism in exon 29 | 41 | Japanese | Schizophrenia | DSM-IV | AIMS & Schooler-Kane criteria | FGAs | PCR-RFLP (*Eco*72I) | No statistically significant genotypic or allele frequencies | (Shinkai et al. 2004) |
| *GSTP1* | rs1695 | 225 | Caucasian and African American | Schizophrenia | DSM-III-R | AIMS & Schooler-Kane criteria | NA | TaqMan allele specific assay | No statistically significant genotypic or allele frequencies | (Shinkai et al. 2005) |
| *GPX1* | rs1050450 | 68 | Caucasian, African American | Schizophrenia | DSM-III-R | AIMS & Schooler-Kane criteria | FGAs | TaqMan allele specific assay | No statistically significant genotypic or allele frequencies | (Shinkai et al. 2006) |
| *SLC6A11, GABRG3 and GABRB2* | rs4684742 (*SLC6A11*), rs2061051 (*GABRG3*), rs918528 (*GABRB2*) | 280 (105 with TD) | Korean | Schizophrenia | DSM-IV | AIMS | FGAs and SGAs | HRM | Association between rs4684742 and TD, as well as gene-gene interactions (*SCL6A11, GABRG3* and *GABRB2*) and TD | (Son et al. 2014) |
| *DRD1, DRD2, DRD3, DRD4, DAT, COMT* | rs5330, rs5331, rs13306309, rs686, rs4532 (DRD1), rs1799732, rs17294542, rs1800497, rs1801028, rs2234689 (DRD2), rs324026,  rs1503670,  rs905568, rs6280  biallelic STR (DRD3),  rs1800955, 120 bp duplication, 48 bp VNTR in exon 3, 120-bp tandem duplication 1.2 kb upstream from initiation codon  (DRD4), 40 bp VNTR (DAT), rs4818, rs4680, rs4633, rs2075507, 900 ins C 3 UTR (COMT) | 335 (96 with TD) | Indian | schizophrenia | DSM-IV | AIMS | FGAs and SGAs | PCR-RFLP | Association between 120 bp duplication marker in DRD4 and TD in genotypic level. Allelic and genotypic association for rs4818 in COMT and significant genotypic association for rs4680 | (Srivastava et al. 2006) |
| *IL10, DBH* | rs1800872 (*IL10*), rs72393728 (5’-Ins/Del in DBH) | 784 (372 with TD) | Han Chinese | Schizophrenia | DSM-IV, PANSS | AIMS | lozapine, risperidone, perphenazine, sulpiride, chlorpromazine, haloperidol, aripiprizol, quetiapine, and other typical and atypical antipsychotics | PCR-RFLP (RsaI) (rs1800872) and agarose gel electrophoresis (rs72393728) | No statistically significant genotypic frequencies | (Sun et al. 2013) |
| *DBH* | rs72393728 (5’-Ins/Del in *DBH*) | 747 (312 with TD) and 625 controls | Han Chinese | Schizophrenia | DSM-IV | AIMS | clozapine, risperidone, quetiapine, aripirazole, olanzapine, loxapine, chlorpromazine, sulpiride, perphenazine, haloperidol, pipotiazine palmitate, and others | agarose gel electrophoresis | No statistically significant genotypic or allele frequencies | (Sun et al. 2013) |
| *HTR2A* | rs6313 | 87 | Chinese (Singaporean) | Schizophrenia | DSM-IV | AIMS | Neuroleptics | PCR-RFLP (Msp) | Association between TT genotype and lower risk of TD, and significant difference in allelic levels between patients with and without TD | (Tan et al. 2001) |
| *CNR1* | rs806368, rs12720071, rs1049353, rs80639, rs806370, rs806374, rs806375  rs806377, rs806378  rs2023239, rs806380, rs806381, rs7752758, rs12528858, rs12205430, rs6914429, rs2180619, rs754387, rs9450902, rs10485170 | 191 (74 with TD) | Caucasian (European) | Schizophrenia or schizoaffective disorder | DSM-III-R or IV | AIMS or modified HSDS | NA | TaqMan assay | Association between rs806374 C allele and CC genotype and TD development and severity | (Tiwari et al. 2012) |
| *ADORA1, ADORA2A, ADORA3* | rs1874142, rs10920568, rs3766566, rs3766560, rs3753472, rs3766553, rs12744240 (*ADORA1*), rs2298383, rs2236624, rs57  51876, rs35320474, rs17004921 (*ADORA2A*), rs3394, rs3393, rs2229155, rs35511654, rs1544223, rs2298191 (*ADORA3*) | 127 | Caucasian (Slovenian) | schizophrenia, schizoaffective disorder and other psychotic disorders | DSM-IV | AIMS | Risperidone, haloperidol, fluphenazine, zuclopenthixol, thioridazine | KASPar allele-specific PCR assay | The CACTAT haplotype of *ADORA3* was associated with TD | (Turčin et al. 2016) |
| *DRD3* | rs6280 | 200 | Caucasian (Scotland, European, German)  Chinese, Korean and Japanese | Schizophrenia | DSM-IV | AIMS & Schooler-Kane criteria | FGAs | TaqMan allele specific  assay | No statistically significant genotypic or allele frequencies | (Utsunomiya et al. 2012) |
| *BDNF* | rs6265 | 815 | NA | Schizophrenia | DSM-IV | AIMS & Schooler-Kane criteria | NA | PCR-RFLP (*Eco*721) | No statistically significant genotypic or allele frequencies | (Wang et al. 2010) |
| *TNFA* | rs1800629 | 760 (350 with TD) | Northern Han Chinese | Schizophrenia | DSM-IV, PANSS | AIMS | clozapine, risperidone perphenazine, sulpiride, chlorpromazine, haloperidol, and other FGAs and SGAs | PCR-RFLP (NcoI) | No statistically significant genotypic or allele frequencies | (Wang et al. 2012) |
| *DRD3* | rs6280 | 59 | Korean | Schizophrenia | DSM-IV | AIMS | Neuroleptic medications | PCR-RFPL (MscI) | Significant association between rs6280 Gly homozygotes and TD | (Woo et al. 2002) |
| *DRD2* | rs4648317, rs1079598, rs2242591, rs2242593, rs1125394, rs2242592,  rs1799732, rs1800497, rs1800498, rs6275, rs1799978, rs6277 | 232 (91 with TD) | Caucasian (European) and African American | schizophrenia or schizoaffective disorder | DSM-III-R or DSM-IV | AIMS, HSDS | FGAs, SGAs | TaqMan allele-specific assays | Association between rs6277 and rs6275 with TD occurrence. rs6277 T allele and rs6275 C allele frequencies were lower in TD patients | (Zai et al. 2007) |
| *AKT1* | rs2498784, rs1130214, rs2494746, rs10149779, rs2494738, rs3730358, rs3803304, rs2494731 | 223 (87 with TD) | Caucasian and African American | schizophrenia | NA | AIMS, HSDS | FGAs, SGAs | NA | No statistically significant genotypic or allele frequencies | (Zai et al. 2008) |
| *DRD4* | rs3758653, rs916457, exon 3 VNTR, rs762502, rs11246226, rs936465 | 171 (70 with TD) | Caucasian (European) | schizophrenia or schizoaffective disorder | DSM-III-R or DSM-IV | AIMS, HSDS | FGAs, SGAs | Illumina SNP platform, high resolution agarose gel (3 VNTR) | Association between the haplotype of rs3758653, rs916457, rs762502, and rs11246226 and TD in males | (Zai et al. 2009a) |
| *DRD3, BDNF* | rs3732782, rs905568, rs7620754, rs7616367, rs7611535, rs1394016, rs9825563, rs1800828, rs6280, rs7633291, rs167770, rs2134655, rs2399496, rs2087017, rs1025398 (DRD3) and rs7934165, rs11030104, rs6265, rs1519480 (BDNF) | 171 (70 with TD) | Caucasian | schizophrenia or schizoaffective disorder | DSM-III-R or DSM-IV | AIMS, HSDS | FGAs, SGAs | Microarray platform (Illumina) | Association between the DRD3 haplotype of rs3732782, rs905568, and rs7620754 with TD, as well as evidence of interaction between BDNF and DRD3 polymorphisms | (Zai et al. 2009b) |
| *NQO1, SOD2* | rs1800566 (*NQO1*), and rs4880 (SOD2) | 223 | Caucasian and African American | Schizophrenia | ΝΑ | AIMS, HSDS | FGAs, SGAs | Assays-on-Demand for rs1800566, PCR-RFLP (BsaWI) for rs4880 | No statistically significant genotypic or allele frequencies | (Zai et al. 2010a) |
| *COMT* | rs737865,  rs6269, rs4633,  rs4818, rs4680, and  rs165599 | 229 (90 with TD) | Caucasian and African American | Schizophrenia, schizoaffective disorder | DSM-III-R or DSM-IV | AIMS or the modified HSDS | NA | Assays-on-Demand (ABI) | Association between AA genotype of rs165599 with TD and a trend of association with higher AIMS scores | (Zai et al. 2010b) |
| *SLC18A2* | rs363224, rs363390, rs1860404,  rs2015586, rs14240, rs363393, rs2072362,  rs2244249, rs363285 | 217 | Caucasian (European) | schizophrenia or schizoaffective disorder | DSM-III-R or DSM-IV | AIMS or the modified HSDS | NA | TaqMan assay | Association between rs363390, rs363224 rs1860404, rs2015586 and rs14240 with TD | (Zai et al. 2013) |
| *HSPG2* | rs2445142 | 217 (87 with TD) | European and African American | Schizophrenia, schizoaffective disorder | DSM-  III-R or DSM-IV | AIMS or HSDS | FGAs, SGAs | TaqMan assay | Significant association between rs2445142 G-allele of *HSPG2* and TD | (Zai et al. 2018) |
| *C4* | copy numbers of C4A, C4B, C4L, C4S and C4AL, C4BL, C4AS, C4BS, C4A, C4B | 129 (48 with TD) | Caucasian (European) | schizophrenia or schizoaffective  disorder | DSM-III-R or DSM-IV | AIMS | FGAs | droplet digital  PCR (ddPCR) and long-range PCR | Nominally significant association between C4BL and TD severity | (Zai et al. 2019b) |
| *NRG1, ERBB4* | rs35753505, rs6994992 (*NRG1*), rs839523 (*ERBB4*) | 153 (66 with TD) | Caucasian (European) | Schizophrenia or schizoaffective dis-order | DSM-IIIR/IV | Schooler and Kane criteria | FGAs and SGAs | TaqMan assay | Association between rs839523 and TD occurrence | (Zai et al. 2019a) |
| *MnSOD* | rs4880 | 101 (42 males with TD) and 50 male controls | Chinese | schizophrenia | DSM IV | AIMS | chlorpromazine, clozapine, fluphenazine, sulpiride, haloperidol and combination | PCR-RFLP (BsaW I) | No statistically significant genotypic or allele frequencies | (Zhang et al. 2002b) |
| *HTR2C* | rs3813929, rs518147 | 42 | Han Chinese | Schizophrenia | DSM-IV | AIMS | Antipsychotics | PCR-RFLP (AciI) | Association between rs518147 and TD | (Zhang et al. 2002a) |
| *BDNF* | rs6265 | 931 (368 with TD) and 546 controls | Han Chinese | Schizophrenia | DSM-IV | AIMS | clozapine, risperidone, quetiapine, aripirazole, olanzapine, loxapine, ziprasidone, chlorpromazine, sulpiride, perphenazine, haloperidol, pipotiazine palmitate | PCR-RFLP (Eco721) | No statistically significant genotypic or allele frequencies | (Zhang et al. 2012) |

*Hillside Simpson Dyskinesia Scale (HSDS), Abnormal Involuntary Movement Scale (AIMS), High resolution melting analysis (HRM), Research Diagnostic Criteria for Tardive Dyskinesia (RDC-TD), limb-truncal dyskinesia (TDlt), Whole exome sequencing (WES), heteroduplex-single strand conformational polymorphism analysis (HEX-SSCP)

Please note that the studies are presented alphabetically, by the first author’s name.

**References**

Al Hadithy AFY, Ivanova SA, Pechlivanoglou P, et al (2009) Tardive dyskinesia and DRD3, HTR2A and HTR2C gene polymorphisms in Russian psychiatric inpatients from Siberia. Prog Neuro-Psychopharmacology Biol Psychiatry 33:475–481. https://doi.org/10.1016/j.pnpbp.2009.01.010

Alkelai A, Greenbaum L, Heinzen EL, et al (2019) New insights into tardive dyskinesia genetics: Implementation of whole-exome sequencing approach. Prog Neuropsychopharmacol Biol Psychiatry 94:109659. https://doi.org/10.1016/j.pnpbp.2019.109659

An H-M, Tan Y-L, Shi J, et al (2013) Extract of Ginkgo biloba is equivalent to vitamin E in attenuating and preventing vacuous chewing movements in a rat model of tardive dyskinesia. Behav Pharmacol 24:610–6. https://doi.org/10.1097/FBP.0b013e3283656d87

An HM, Tan YL, Shi J, et al (2016a) Beneficial effects of EGb761 and vitamin E on haloperidol-induced vacuous chewing movements in rats: Possible involvement of S100B mechanisms. Behav Brain Res 297:124–30. https://doi.org/10.1016/j.bbr.2015.10.004

An HM, Tan YL, Shi J, et al (2016b) Ginkgo biloba leaf extract and alpha-tocopherol attenuate haloperidol-induced orofacial dyskinesia in rats: Possible implication of antiapoptotic mechanisms by preventing Bcl-2 decrease and Bax elevation. Phytomedicine 23:1653–1660. https://doi.org/10.1016/j.phymed.2016.10.009

Andreassen OA, MacEwan T, Gulbrandsen AK, et al (1997) Non-functional CYP2D6 alleles and risk for neuroleptic-induced movement disorders in schizophrenic patients. Psychopharmacology (Berl) 131:174–9

Armstrong M, Daly AK, Blennerhassett R, et al (1997) Antipsychotic drug-induced movement disorders in schizophrenics in relation to CYP2D6 genotype. Br J Psychiatry 170:23–6

Arthur H, Dahl ML, Siwers B, Sjöqvist F (1995) Polymorphic drug metabolism in schizophrenic patients with tardive dyskinesia. J Clin Psychopharmacol 15:211–6

Bakker PR, Al Hadithy AFY, Amin N, et al (2012) Antipsychotic-induced movement disorders in long-stay psychiatric patients and 45 tag SNPs in 7 candidate genes: a prospective study. PLoS One 7:e50970. https://doi.org/10.1371/journal.pone.0050970

Basile VS, Masellis M, Badri F, et al (1999) Association of the MscI polymorphism of the dopamine D3 receptor gene with tardive dyskinesia in schizophrenia. Neuropsychopharmacology 21:17–27. https://doi.org/10.1016/S0893-133X(98)00114-6

Basile VS, Ozdemir V, Masellis M, et al (2000) A functional polymorphism of the cytochrome P450 1A2 (CYP1A2) gene: association with tardive dyskinesia in schizophrenia. Mol Psychiatry 5:410–7

Basile VS, Ozdemir V, Masellis M, et al (2001) Lack of association between serotonin-2A receptor gene (HTR2A) polymorphisms and tardive dyskinesia in schizophrenia. Mol Psychiatry 6:230–4. https://doi.org/10.1038/sj.mp.4000847

Bishnoi M, Boparai RK (2012) An animal model to study the molecular basis of tardive dyskinesia. Methods Mol Biol 829:193–201. https://doi.org/10.1007/978-1-61779-458-2_12

Boiko AS, Ivanova SA, Pozhidaev I V, et al (2020) Pharmacogenetics of tardive dyskinesia in schizophrenia: The role of CHRM1 and CHRM2 muscarinic receptors. World J Biol Psychiatry 21:72–77. https://doi.org/10.1080/15622975.2018.1548780

Boke O, Gunes S, Kara N, et al (2007) Association of serotonin 2A receptor and lack of association of CYP1A2 gene polymorphism with tardive dyskinesia in a Turkish population. DNA Cell Biol 26:527–31. https://doi.org/10.1089/dna.2007.0605

Bordia T, McIntosh JM, Quik M (2012) Nicotine reduces antipsychotic-induced orofacial dyskinesia in rats. J Pharmacol Exp Ther 340:612–9. https://doi.org/10.1124/jpet.111.189100

Bošković M, Vovk T, Saje M, et al (2013) Association of SOD2, GPX1, CAT, and TNF genetic polymorphisms with oxidative stress, neurochemistry, psychopathology, and extrapyramidal symptoms in schizophrenia. Neurochem Res 38:433–42. https://doi.org/10.1007/s11064-012-0937-4

Brockmöller J, Kirchheiner J, Schmider J, et al (2002) The impact of the CYP2D6 polymorphism on haloperidol pharmacokinetics and on the outcome of haloperidol treatment. Clin Pharmacol Ther 72:438–52. https://doi.org/10.1067/mcp.2002.127494

Busanello A, Leal CQ, Peroza LR, et al (2017) Resveratrol Protects Against Vacuous Chewing Movements Induced by Chronic Treatment with Fluphenazine. Neurochem Res 42:3033–3040. https://doi.org/10.1007/s11064-017-2335-4

Busanello A, Peroza LR, Wagner C, et al (2012) Resveratrol reduces vacuous chewing movements induced by acute treatment with fluphenazine. Pharmacol Biochem Behav 101:307–10. https://doi.org/10.1016/j.pbb.2012.01.007

Ceretta APC, de Freitas CM, Schaffer LF, et al (2018) Gabapentin reduces haloperidol-induced vacuous chewing movements in mice. Pharmacol Biochem Behav 166:21–26. https://doi.org/10.1016/j.pbb.2018.01.003

Chen C-N, Chang K-C, Wang M-H, et al (2018) Protective Effect of L-Theanine on Haloperidol-Induced Orofacial. Chin J Physiol 61:35–41. https://doi.org/10.4077/CJP.2018.BAG529

Chen CH, Wei FC, Koong FJ, Hsiao KJ (1997) Association of TaqI A polymorphism of dopamine D2 receptor gene and tardive dyskinesia in schizophrenia. Biol Psychiatry 41:827–9. https://doi.org/10.1016/S0006-3223(96)00543-4

Chong S-A, Tan E-C, Tan CH, et al (2003a) Polymorphisms of dopamine receptors and tardive dyskinesia among Chinese patients with schizophrenia. Am J Med Genet B Neuropsychiatr Genet 116B:51–4. https://doi.org/10.1002/ajmg.b.10004

Chong S-A, Tan E-C, Tan CH, Mythily (2003b) Smoking and tardive dyskinesia: lack of involvement of the CYP1A2 gene. J Psychiatry Neurosci 28:185–9

Chong SA, Tan EC, Tan CH, et al (2000) Tardive dyskinesia is not associated with the serotonin gene polymorphism (5-HTTLPR) in Chinese. Am J Med Genet 96:712–5

Creed MC, Hamani C, Nobrega JN (2012) Early gene mapping after deep brain stimulation in a rat model of tardive dyskinesia: comparison with transient local inactivation. Eur Neuropsychopharmacol 22:506–17. https://doi.org/10.1016/j.euroneuro.2011.11.004

Crowley JJ, Adkins DE, Pratt AL, et al (2012a) Antipsychotic-induced vacuous chewing movements and extrapyramidal side effects are highly heritable in mice. Pharmacogenomics J 12:147–55. https://doi.org/10.1038/tpj.2010.82

Crowley JJ, Kim Y, Szatkiewicz JP, et al (2012b) Genome-wide association mapping of loci for antipsychotic-induced extrapyramidal symptoms in mice. Mamm Genome 23:322–35. https://doi.org/10.1007/s00335-011-9385-8

Datta S, Jamwal S, Deshmukh R, Kumar P (2016) Beneficial effects of lycopene against haloperidol induced orofacial dyskinesia in rats: Possible neurotransmitters and neuroinflammation modulation. Eur J Pharmacol 771:229–35. https://doi.org/10.1016/j.ejphar.2015.12.032

de Leon J, Susce MT, Pan R-M, et al (2005) Polymorphic variations in GSTM1, GSTT1, PgP, CYP2D6, CYP3A5, and dopamine D2 and D3 receptors and their association with tardive dyskinesia in severe mental illness. J Clin Psychopharmacol 25:448–56

De Luca V, Souza RP, Viggiano E, et al (2009) MDR1 gene in tardive dyskinesia scale scores: Comparison of strategies for quantitative trait haplotype analysis. Schizophr. Res. 110

de Oliveira GV, Gomes PXL, de Araújo FYR, et al (2013) Prevention of haloperidol-induced alterations in brain acetylcholinesterase activity by vitamins B co-administration in a rodent model of tardive dyskinesia. Metab Brain Dis 28:53–9. https://doi.org/10.1007/s11011-012-9345-3

Dhingra D, Goswami S, Gahalain N (2018) Protective effect of hesperetin against haloperidol-induced orofacial dyskinesia and catalepsy in rats. Nutr Neurosci 21:667–675. https://doi.org/10.1080/1028415X.2017.1338549

Ellingrod VL, Schultz SK, Arndt S (2002) Abnormal movements and tardive dyskinesia in smokers and nonsmokers with schizophrenia genotyped for cytochrome P450 2D6. Pharmacotherapy 22:1416–9

Fedorenko OY, Loonen AJM, Lang F, et al (2014) Association study indicates a protective role of phosphatidylinositol-4-phosphate-5-kinase against tardive dyskinesia. Int J Neuropsychopharmacol 18:. https://doi.org/10.1093/ijnp/pyu098

Fu Y, Fan C, Deng H, et al (2006) Association of CYP2D6 and CYP1A2 gene polymorphism with tardive dyskinesia in Chinese schizophrenic patients. Acta Pharmacol Sin 27:328–32. https://doi.org/10.1111/j.1745-7254.2006.00279.x

Gaitonde EJ, Morris A, Sivagnanasundaram S, et al (1996) Assessment of association of D3 dopamine receptor MscI polymorphism with schizophrenia: analysis of symptom ratings, family history, age at onset, and movement disorders. Am J Med Genet 67:455–8. https://doi.org/10.1002/(SICI)1096-8628(19960920)67:5<455::AID-AJMG3>3.0.CO;2-J

Garcia-Barceló MM, Lam LC, Ungvari GS, et al (2001) Dopamine D3 receptor gene and tardive dyskinesia in Chinese schizophrenic patients. J Neural Transm 108:671–7. https://doi.org/10.1007/s007020170044

Greenbaum L, Alkelai A, Zozulinsky P, et al (2012) Support for association of HSPG2 with tardive dyskinesia in Caucasian populations. Pharmacogenomics J 12:513–20. https://doi.org/10.1038/tpj.2011.32

Grossman I, Sullivan PF, Walley N, et al (2008) Genetic determinants of variable metabolism have little impact on the clinical use of leading antipsychotics in the CATIE study. Genet Med 10:720–9. https://doi.org/10.1097/GIM.0b013e3181863239

Grover S, Kumar P, Singh K, et al (2013) Possible beneficial effect of peroxisome proliferator-activated receptor (PPAR)--α and γ agonist against a rat model of oral dyskinesia. Pharmacol Biochem Behav 111:17–23. https://doi.org/10.1016/j.pbb.2013.08.001

Herken H, Erdal ME, Böke O, Savaş HA (2003) Tardive dyskinesia is not associated with the polymorphisms of 5-HT2A receptor gene, serotonin transporter gene and catechol-o-methyltransferase gene. Eur Psychiatry 18:77–81

Hernandez G, Mahmoudi S, Cyr M, et al (2019) Tardive dyskinesia is associated with altered putamen Akt/GSK-3β signaling in nonhuman primates. Mov Disord 34:717–726. https://doi.org/10.1002/mds.27630

Hitzeroth A, Niehaus DJH, Koen L, et al (2007) Association between the MnSOD Ala-9Val polymorphism and development of schizophrenia and abnormal involuntary movements in the Xhosa population. Prog Neuropsychopharmacol Biol Psychiatry 31:664–72. https://doi.org/10.1016/j.pnpbp.2006.12.019

Hori H, Ohmori O, Shinkai T, et al (2001) Association between three functional polymorphisms of dopamine D2 receptor gene and tardive dyskinesia in schizophrenia. Am J Med Genet 105:774–8

Hori H, Ohmori O, Shinkai T, et al (2000) Manganese superoxide dismutase gene polymorphism and schizophrenia: relation to tardive dyskinesia. Neuropsychopharmacology 23:170–7. https://doi.org/10.1016/S0893-133X(99)00156-6

Hsieh C-J, Chen Y-C, Lai M-S, et al (2011) Genetic variability in serotonin receptor and transporter genes may influence risk for tardive dyskinesia in chronic schizophrenia. Psychiatry Res 188:175–6. https://doi.org/10.1016/j.psychres.2010.10.006

Hui L, Han M, Huang XF, et al (2015) Possible association between DBH 19 bp insertion/deletion polymorphism and clinical symptoms in schizophrenia with tardive dyskinesia. J Neural Transm 122:907–14. https://doi.org/10.1007/s00702-014-1327-7

Hui L, Han M, Yin GZ, et al (2017) Association between DBH 19bp insertion/deletion polymorphism and cognition in schizophrenia with and without tardive dyskinesia. Schizophr Res 182:104–109. https://doi.org/10.1016/j.schres.2016.10.028

Inada T, Senoo H, Iijima Y, et al (2003) Cytochrome P450 II D6 gene polymorphisms and the neuroleptic-induced extrapyramidal symptoms in Japanese schizophrenic patients. Psychiatr Genet 13:163–8. https://doi.org/10.1097/00041444-200309000-00005

Ivanova SA, Al Hadithy AFY, Brazovskaya N, et al (2012a) No involvement of the adenosine A2A receptor in tardive dyskinesia in Russian psychiatric inpatients from Siberia. Hum Psychopharmacol 27:334–7. https://doi.org/10.1002/hup.2226

Ivanova SA, Filipenko ML, Vyalova NM, et al (2016a) CYP1A2 and CYP2D6 Gene Polymorphisms in Schizophrenic Patients with Neuroleptic Drug-Induced Side Effects. Bull Exp Biol Med 160:687–90. https://doi.org/10.1007/s10517-016-3250-4

Ivanova SA, Geers LM, Al Hadithy AFY, et al (2014) Dehydroepiandrosterone sulphate as a putative protective factor against tardive dyskinesia. Prog Neuropsychopharmacol Biol Psychiatry 50:172–7. https://doi.org/10.1016/j.pnpbp.2013.12.015

Ivanova SA, Loonen AJ, Bakker PR, et al (2016b) Likelihood of mechanistic roles for dopaminergic, serotonergic and glutamatergic receptors in tardive dyskinesia: A comparison of genetic variants in two independent patient populations. SAGE Open Med 4:205031211664367. https://doi.org/10.1177/2050312116643673

Ivanova SA, Loonen AJM, Pechlivanoglou P, et al (2012b) NMDA receptor genotypes associated with the vulnerability to develop dyskinesia. Transl Psychiatry 2:e67. https://doi.org/10.1038/tp.2011.66

Ivanova SA, Toshchakova VA, Filipenko ML, et al (2015) Cytochrome P450 1A2 co-determines neuroleptic load and may diminish tardive dyskinesia by increased inducibility. World J Biol Psychiatry 16:200–5. https://doi.org/10.3109/15622975.2014.995222

Jaanson P, Marandi T, Kiivet R-A, et al (2002) Maintenance therapy with zuclopenthixol decanoate: associations between plasma concentrations, neurological side effects and CYP2D6 genotype. Psychopharmacology (Berl) 162:67–73. https://doi.org/10.1007/s00213-002-1059-5

Kaiser R, Tremblay P-B, Klufmöller F, et al (2002) Relationship between adverse effects of antipsychotic treatment and dopamine D(2) receptor polymorphisms in patients with schizophrenia. Mol Psychiatry 7:695–705. https://doi.org/10.1038/sj.mp.4001054

Kajero JA, Seedat S, Ohaeri J, et al (2020) Investigation of the effects of cannabidiol on vacuous chewing movements, locomotion, oxidative stress and blood glucose in rats treated with oral haloperidol. World J Biol Psychiatry 21:612–626. https://doi.org/10.1080/15622975.2020.1752934

Kang S-G, Lee H-J, Yoon H-K, et al (2013) There is no evidence for an association between the serotonin receptor 3A gene C178T polymorphism and tardive dyskinesia in Korean schizophrenia patients. Nord J Psychiatry 67:214–8. https://doi.org/10.3109/08039488.2012.732114

Kapitany T, Meszaros K, Lenzinger E, et al (1998) Genetic polymorphisms for drug metabolism (CYP2D6) and tardive dyskinesia in schizophrenia. Schizophr Res 32:101–6

Khan MM, Xiao J, Hollingsworth TJ, et al (2019) Gnal haploinsufficiency causes genomic instability and increased sensitivity to haloperidol. Exp Neurol 318:61–70. https://doi.org/10.1016/j.expneurol.2019.04.014

Kim I-S, Yoon H-K, Kang S-G, et al (2012) No association between PAWR gene polymorphisms and tardive dyskinesia in schizophrenia patients. Psychiatry Investig 9:191–4. https://doi.org/10.4306/pi.2012.9.2.191

Kimura T, Shono M, Yokota S, et al (2000) Apolipoprotein E epsilon4 and tardive dyskinesia in a Japanese population. J Psychiatr Res 34:329–32. https://doi.org/10.1016/s0022-3956(00)00023-6

Koning JP, Vehof J, Burger H, et al (2012) Association of two DRD2 gene polymorphisms with acute and tardive antipsychotic-induced movement disorders in young Caucasian patients. Psychopharmacology (Berl) 219:727–36. https://doi.org/10.1007/s00213-011-2394-1

Koola MM, Tsapakis EM, Wright P, et al (2014) Association of tardive dyskinesia with variation in CYP2D6: Is there a role for active metabolites? J Psychopharmacol 28:665–70. https://doi.org/10.1177/0269881114523861

Kronbauer M, Metz VG, Roversi K, et al (2017) Influence of magnesium supplementation on movement side effects related to typical antipsychotic treatment in rats. Behav Brain Res 320:400–411. https://doi.org/10.1016/j.bbr.2016.10.049

Lai I-C, Chen M-L, Wang Y-C, et al (2011a) Analysis of genetic variations in the human melatonin receptor (MTNR1A, MTNR1B) genes and antipsychotics-induced tardive dyskinesia in schizophrenia. World J Biol Psychiatry 12:143–8. https://doi.org/10.3109/15622975.2010.496870

Lai I-C, Liao D-L, Bai Y-M, et al (2002) Association study of the estrogen receptor polymorphisms with tardive dyskinesia in schizophrenia. Neuropsychobiology 46:173–5. https://doi.org/10.1159/000067808

Lai I-C, Mo G-H, Chen M-L, et al (2011b) Analysis of genetic variations in the dopamine D1 receptor (DRD1) gene and antipsychotics-induced tardive dyskinesia in schizophrenia. Eur J Clin Pharmacol 67:383–388. https://doi.org/10.1007/s00228-010-0967-2

Lam LC, Garcia-Barcelo MM, Ungvari GS, et al (2001) Cytochrome P450 2D6 genotyping and association with tardive dyskinesia in Chinese schizophrenic patients. Pharmacopsychiatry 34:238–41. https://doi.org/10.1055/s-2001-18035

Lanning R, Lett TA, Tiwari AK, et al (2017) Association study between the neurexin-1 gene and tardive dyskinesia. Hum Psychopharmacol 32:. https://doi.org/10.1002/hup.2568

Lerer B, Segman RH, Fangerau H, et al (2002) Pharmacogenetics of tardive dyskinesia: combined analysis of 780 patients supports association with dopamine D3 receptor gene Ser9Gly polymorphism. Neuropsychopharmacology 27:105–19. https://doi.org/10.1016/S0893-133X(02)00293-2

Levchenko A, Vyalova N, Pozhidaev I V, et al (2019) No evidence so far of a major role of AKT1 and GSK3B in the pathogenesis of antipsychotic-induced tardive dyskinesia. https://doi.org/10.1002/hup.2685

Lévesque C, Hernandez G, Mahmoudi S, et al (2017) Deficient striatal adaptation in aminergic and glutamatergic neurotransmission is associated with tardive dyskinesia in non-human primates exposed to antipsychotic drugs. Neuroscience 361:43–57. https://doi.org/10.1016/j.neuroscience.2017.07.068

Li H, Tan Y, Wang Z, et al (2013) Association study on tardive dyskinesia and polymorphisms in COMT and MAOA in Chinese population. Psychiatr Genet 23:176. https://doi.org/10.1097/YPG.0b013e32835e8df6

Liao DL, Yeh YC, Chen HM, et al (2001) Association between the Ser9Gly polymorphism of the dopamine D3 receptor gene and tardive dyskinesia in Chinese schizophrenic patients. Neuropsychobiology 44:95–8. https://doi.org/10.1159/000054924

Liou Y-J, Lai I-C, Liao D-L, et al (2006) The human dopamine receptor D2 (DRD2) gene is associated with tardive dyskinesia in patients with schizophrenia. Schizophr Res 86:323–325. https://doi.org/10.1016/j.schres.2006.04.008

Liou Y-J, Wang Y-C, Bai Y-M, et al (2004) Cytochrome P-450 2D6*10 C188T polymorphism is associated with antipsychotic-induced persistent tardive dyskinesia in Chinese schizophrenic patients. Neuropsychobiology 49:167–73. https://doi.org/10.1159/000077360

Lohmann PL, Bagli M, Krauss H, et al (2003) CYP2D6 polymorphism and tardive dyskinesia in schizophrenic patients. Pharmacopsychiatry 36:73–8. https://doi.org/10.1055/s-2003-39048

Løvlie R, Daly AK, Blennerhassett R, et al (2000) Homozygosity for the Gly-9 variant of the dopamine D3 receptor and risk for tardive dyskinesia in schizophrenic patients. Int J Neuropsychopharmacol 3:61–65. https://doi.org/10.1017/S1461145700001796

Lu JY, Tiwari AK, Freeman N, et al (2020) Liver enzyme CYP2D6 gene and tardive dyskinesia. Pharmacogenomics 21:1065–1072. https://doi.org/10.2217/pgs-2020-0065

Lu JY, Tiwari AK, Zai GC, et al (2018) Association study of Disrupted-In-Schizophrenia-1 gene variants and tardive dyskinesia. Neurosci Lett 686:17–22. https://doi.org/10.1016/j.neulet.2018.08.007

Maes MS, Lu JY, Tiwari AK, et al (2021) Schizophrenia-associated gene dysbindin-1 and tardive dyskinesia. Drug Dev Res 82:678–684. https://doi.org/10.1002/ddr.21681

Mahmoudi S, Blanchet PJ, Lévesque D (2013) Haloperidol-induced striatal Nur77 expression in a non-human primate model of tardive dyskinesia. Eur J Neurosci 38:2192–8. https://doi.org/10.1111/ejn.12198

Mahmoudi S, Lévesque D, Blanchet PJ (2014) Upregulation of dopamine D3, not D2, receptors correlates with tardive dyskinesia in a primate model. Mov Disord 29:1125–1133. https://doi.org/10.1002/mds.25909

Matsumoto C, Ohmori O, Shinkai T, et al (2004a) Genetic association analysis of functional polymorphisms in the cytochrome P450 1A2 (CYP1A2) gene with tardive dyskinesia in Japanese patients with schizophrenia. Psychiatr Genet 14:209–13

Matsumoto C, Shinkai T, Hori H, et al (2004b) Polymorphisms of dopamine degradation enzyme (COMT and MAO) genes and tardive dyskinesia in patients with schizophrenia. Psychiatry Res 127:1–7. https://doi.org/10.1016/j.psychres.2004.03.011

Miksys S, Wadji FB, Tolledo EC, et al (2017) Rat brain CYP2D enzymatic metabolism alters acute and chronic haloperidol side-effects by different mechanisms. Prog Neuropsychopharmacol Biol Psychiatry 78:140–148. https://doi.org/10.1016/j.pnpbp.2017.04.030

Nade VS, Shendye N V, Kawale LA, et al (2013) Protective effect of nebivolol on reserpine-induced neurobehavioral and biochemical alterations in rats. Neurochem Int 63:316–21. https://doi.org/10.1016/j.neuint.2013.07.002

Nagaoka K, Nagashima T, Asaoka N, et al (2021) Striatal TRPV1 activation by acetaminophen ameliorates dopamine D2 receptor antagonist-induced orofacial dyskinesia. JCI insight 6:. https://doi.org/10.1172/jci.insight.145632

Nikoloff D, Shim JC, Fairchild M, et al (2002) Association between CYP2D6 genotype and tardive dyskinesia in Korean schizophrenics. Pharmacogenomics J 2:400–7. https://doi.org/10.1038/sj.tpj.6500138

Ohmori, Kojima, Shinkai, et al (1999) Genetic association analysis between CYP2D6*2 allele and tardive dyskinesia in schizophrenic patients. Psychiatry Res 87:239–44

Ohmori O, Shinkai T, Hori H, et al (2001) Polymorphisms of mu and delta opioid receptor genes and tardive dyskinesia in patients with schizophrenia. Schizophr Res 52:137–8. https://doi.org/10.1016/s0920-9964(00)00188-2

Ohmori O, Shinkai T, Hori H, Nakamura J (2002) Genetic association analysis of 5-HT(6) receptor gene polymorphism (267C/T) with tardive dyskinesia. Psychiatry Res 110:97–102

Ohmori O, Suzuki T, Kojima H, et al (1998) Tardive dyskinesia and debrisoquine 4-hydroxylase (CYP2D6) genotype in Japanese schizophrenics. Schizophr Res 32:107–13

Oliveira PA de, Dalton JAR, López-Cano M, et al (2017) Angiotensin II type 1/adenosine A 2A receptor oligomers: a novel target for tardive dyskinesia. Sci Rep 7:1857. https://doi.org/10.1038/s41598-017-02037-z

Park Y-M, Kang S-G, Choi J-E, et al (2011) No Evidence for an Association between Dopamine D2 Receptor Polymorphisms and Tardive Dyskinesia in Korean Schizophrenia Patients. Psychiatry Investig 8:49–54. https://doi.org/10.4306/pi.2011.8.1.49

Patil RA, Hiray YA, Kasture SB (2012) Reversal of reserpine-induced orofacial dyskinesia and catalepsy by Nardostachys jatamansi. Indian J Pharmacol 44:340–4. https://doi.org/10.4103/0253-7613.96307

Peroza LR, Schaffer LF, De Freitas CM, et al (2016) Alteration of Cytokines Levels in the Striatum of Rats: Possible Participation in Vacuous Chewing Movements Induced by Antipsycotics. Neurochem Res 41:2481–9. https://doi.org/10.1007/s11064-016-1961-6

Plesnicar BK, Zalar B, Breskvar K, Dolzan V (2006) The influence of the CYP2D6 polymorphism on psychopathological and extrapyramidal symptoms in the patients on long-term antipsychotic treatment. J Psychopharmacol 20:829–33. https://doi.org/10.1177/0269881106062894

Pozhidaev I V, Paderina DZ, Fedorenko OY, et al (2020) 5-Hydroxytryptamine Receptors and Tardive Dyskinesia in Schizophrenia. Front Mol Neurosci 13:63. https://doi.org/10.3389/fnmol.2020.00063

Reinheimer JB, Bressan GN, de Freitas CM, et al (2020) Effects of CATECHIN on reserpine-induced vacuous chewing movements: behavioral and biochemical analysis. Naunyn Schmiedebergs Arch Pharmacol 393:2439–2452. https://doi.org/10.1007/s00210-020-01923-0

Rietschel M, Krauss H, Müller DJ, et al (2000) Dopamine D3 receptor variant and tardive dyskinesia. Eur Arch Psychiatry Clin Neurosci 250:31–5

Röpke J, Busanello A, Leal CQ, et al (2014) Anandamide attenuates haloperidol-induced vacuous chewing movements in rats. Prog Neuropsychopharmacol Biol Psychiatry 54:195–9. https://doi.org/10.1016/j.pnpbp.2014.04.006

Röpke J, Ferreira-Vieira TH, Iglesias LP, et al (2021) Protective role of endocannabinoid signaling in an animal model of haloperidol-induced tardive dyskinesia. Pharmacol Biochem Behav 206:173193. https://doi.org/10.1016/j.pbb.2021.173193

Samad N, Haleem DJ (2017) Antioxidant effects of rice bran oil mitigate repeated haloperidol-induced tardive dyskinesia in male rats. Metab Brain Dis 32:1099–1107. https://doi.org/10.1007/s11011-017-0002-8

Schaffer LF, de Freitas CM, Chiapinotto Ceretta AP, et al (2016) Harpagophytum Procumbens Ethyl Acetate Fraction Reduces Fluphenazine-Induced Vacuous Chewing Movements and Oxidative Stress in Rat Brain. Neurochem Res 41:1170–84. https://doi.org/10.1007/s11064-015-1811-y

Schulze TG, Schumacher J, Müller DJ, et al (2001) Lack of association between a functional polymorphism of the cytochrome P450 1A2 (CYP1A2) gene and tardive dyskinesia in schizophrenia. Am J Med Genet 105:498–501

Scordo MG, Spina E, Romeo P, et al (2000) CYP2D6 genotype and antipsychotic-induced extrapyramidal side effects in schizophrenic patients. Eur J Clin Pharmacol 56:679–83. https://doi.org/10.1007/s002280000222

Segman R, Neeman T, Heresco-Levy U, et al (1999) Genotypic association between the dopamine D3 receptor and tardive dyskinesia in chronic schizophrenia. Mol Psychiatry 4:247–53

Segman RH, Goltser T, Heresco-Levy U, et al (2003) Association of dopaminergic and serotonergic genes with tardive dyskinesia in patients with chronic schizophrenia. Pharmacogenomics J 3:277–83. https://doi.org/10.1038/sj.tpj.6500194

Segman RH, Heresco-Levy U, Finkel B, et al (2000) Association between the serotonin 2C receptor gene and tardive dyskinesia in chronic schizophrenia: additive contribution of 5-HT2Cser and DRD3gly alleles to susceptibility. Psychopharmacology (Berl) 152:408–13

Segman RH, Heresco-Levy U, Finkel B, et al (2001) Association between the serotonin 2A receptor gene and tardive dyskinesia in chronic schizophrenia. Mol Psychiatry 6:225–9. https://doi.org/10.1038/sj.mp.4000842

Segman RH, Heresco-Levy U, Yakir A, et al (2002a) Interactive effect of cytochrome P450 17alpha-hydroxylase and dopamine D3 receptor gene polymorphisms on abnormal involuntary movements in chronic schizophrenia. Biol Psychiatry 51:261–3. https://doi.org/10.1016/s0006-3223(01)01302-6

Segman RH, Shapira Y, Modai I, et al (2002b) Angiotensin converting enzyme gene insertion/deletion polymorphism: case-control association studies in schizophrenia, major affective disorder, and tardive dyskinesia and a family-based association study in schizophrenia. Am J Med Genet 114:310–4. https://doi.org/10.1002/ajmg.10255

Sekiguchi K, Kanno H, Yamaguchi T, et al (2012) Ameliorative effect of yokukansan on vacuous chewing movement in haloperidol-induced rat tardive dyskinesia model and involvement of glutamatergic system. Brain Res Bull 89:151–8. https://doi.org/10.1016/j.brainresbull.2012.08.008

Shi J, Tan YL, Wang ZR, et al (2016) Ginkgo biloba and vitamin E ameliorate haloperidol-induced vacuous chewingmovement and brain-derived neurotrophic factor expression in a rat tardive dyskinesia model. Pharmacol Biochem Behav 148:53–8. https://doi.org/10.1016/j.pbb.2016.06.003

Shinkai T, De Luca V, Hwang R, et al (2005) Association study between a functional glutathione S-transferase (GSTP1) gene polymorphism (Ile105Val) and tardive dyskinesia. Neurosci Lett 388:116–20. https://doi.org/10.1016/j.neulet.2005.06.038

Shinkai T, Müller DJ, De Luca V, et al (2006) Genetic association analysis of the glutathione peroxidase (GPX1) gene polymorphism (Pro197Leu) with tardive dyskinesia. Psychiatry Res 141:123–8. https://doi.org/10.1016/j.psychres.2004.06.023

Shinkai T, Ohmori O, Matsumoto C, et al (2004) Genetic association analysis of neuronal nitric oxide synthase gene polymorphism with tardive dyskinesia. Neuromolecular Med 5:163–70. https://doi.org/10.1385/NMM:5:2:163

Son W-Y, Lee H-J, Yoon H-K, et al (2014) Gaba transporter SLC6A11 gene polymorphism associated with tardive dyskinesia. Nord J Psychiatry 68:123–8. https://doi.org/10.3109/08039488.2013.780260

Sonego AB, Prado DS, Vale GT, et al (2018) Cannabidiol prevents haloperidol-induced vacuos chewing movements and inflammatory changes in mice via PPARγ receptors. Brain Behav Immun 74:241–251. https://doi.org/10.1016/j.bbi.2018.09.014

Soung H-S, Wang M-H, Chang K-C, et al (2018) L-Theanine Decreases Orofacial Dyskinesia Induced by Reserpine in Rats. Neurotox Res 34:375–387. https://doi.org/10.1007/s12640-018-9897-z

Srivastava V, Varma PG, Prasad S, et al (2006) Genetic susceptibility to tardive dyskinesia among schizophrenia subjects: IV. Role of dopaminergic pathway gene polymorphisms. Pharmacogenet Genomics 16:111–7. https://doi.org/10.1097/01.fpc.0000184957.98150.0f

Steen VM, Løvlie R, MacEwan T, McCreadie RG (1997) Dopamine D3-receptor gene variant and susceptibility to tardive dyskinesia in schizophrenic patients. Mol Psychiatry 2:139–45

Sun H, Wang F, Fan H, et al (2013) The interaction of polymorphisms of IL10 and DBH was associated with general symptoms of PANSS with TD in Chinese Han schizophrenic patients. PLoS One 8:e70963. https://doi.org/10.1371/journal.pone.0070963

Tan EC, Chong SA, Mahendran R, et al (2001) Susceptibility to neuroleptic-induced tardive dyskinesia and the T102C polymorphism in the serotonin type 2A receptor. Biol Psychiatry 50:144–7

Thakur KS, Prakash A, Bisht R, Bansal PK (2015) Beneficial effect of candesartan and lisinopril against haloperidol-induced tardive dyskinesia in rat. J Renin Angiotensin Aldosterone Syst 16:917–29. https://doi.org/10.1177/1470320313515038

Tiwari AK, Deshpande SN, Rao AR, et al (2005a) Genetic susceptibility to tardive dyskinesia in chronic schizophrenia subjects: I. Association of CYP1A2 gene polymorphism. Pharmacogenomics J 5:60–9. https://doi.org/10.1038/sj.tpj.6500282

Tiwari AK, Deshpande SN, Rao AR, et al (2005b) Genetic susceptibility to tardive dyskinesia in chronic schizophrenia subjects: III. Lack of association of CYP3A4 and CYP2D6 gene polymorphisms. Schizophr Res 75:21–6. https://doi.org/10.1016/j.schres.2004.12.011

Tiwari AK, Zai CC, Likhodi O, et al (2012) Association study of cannabinoid receptor 1 (CNR1) gene in tardive dyskinesia. Pharmacogenomics J 12:260–6. https://doi.org/10.1038/tpj.2010.93

Tsai C-C, Wang M-H, Chang K-C, et al (2019) Possible nitric oxide mechanism involved in the protective effect of L-theanine on haloperidol-induced orofacial dyskinesia. Chin J Physiol 62:17–26. https://doi.org/10.4103/CJP.CJP_8_19

Turčin A, Dolžan V, Porcelli S, et al (2016) Adenosine Hypothesis of Antipsychotic Drugs Revisited: Pharmacogenomics Variation in Nonacute Schizophrenia. OMICS 20:283–9. https://doi.org/10.1089/omi.2016.0003

Utsunomiya K, Shinkai T, Sakata S, et al (2012) Genetic association between the dopamine D3 receptor gene polymorphism (Ser9Gly) and tardive dyskinesia in patients with schizophrenia: a reevaluation in East Asian populations. Neurosci Lett 507:52–6. https://doi.org/10.1016/j.neulet.2011.11.050

Wang F, Fan H, Sun H, et al (2012) Association between TNF-α promoter -308A/G polymorphism and tardive dyskinesian Chinese Han patients with schizophrenia. Prog Neuropsychopharmacol Biol Psychiatry 37:106–10. https://doi.org/10.1016/j.pnpbp.2011.12.007

Wang M-H, Lin R-F, Tseng H-C, et al (2015) (-) Epigallocatechin-3-gallate attenuates reserpine-induced orofacial dyskinesia and oxidative stress in rat striatum. Pharmacol Biochem Behav 131:71–6. https://doi.org/10.1016/j.pbb.2015.02.003

Wang M-H, Yang C-C, Tseng H-C, et al (2021) Naringin Ameliorates Haloperidol-Induced Neurotoxicity and Orofacial Dyskinesia in a Rat Model of Human Tardive Dyskinesia. Neurotox Res 39:774–786. https://doi.org/10.1007/s12640-021-00333-1

Wang Y, Dong Wang J, Ran Wu H, et al (2010) The Val66Met polymorphism of the brain-derived neurotrophic factor gene is not associated with risk for schizophrenia and tardive dyskinesia in Han Chinese population. Schizophr Res 120:240–242. https://doi.org/10.1016/j.schres.2010.03.020

Woo S, Kim JW, Rha E, et al (2002) Association of the Ser9Gly polymorphism in the dopamine D3 receptor gene with tardive dyskinesia in Korean schizophrenics. Psychiatry Clin Neurosci 56:469–74. https://doi.org/10.1046/j.1440-1819.2002.01038.x

Zai CC, Hwang RW, De Luca V, et al (2007) Association study of tardive dyskinesia and twelve DRD2 polymorphisms in schizophrenia patients. Int J Neuropsychopharmacol 10:639–51. https://doi.org/10.1017/S1461145706007152

Zai CC, Lee FH, Tiwari AK, et al (2018) Investigation of the HSPG2 Gene in Tardive Dyskinesia - New Data and Meta-Analysis. Front Pharmacol 9:974. https://doi.org/10.3389/fphar.2018.00974

Zai CC, Romano-Silva MA, Hwang R, et al (2008) Genetic study of eight AKT1 gene polymorphisms and their interaction with DRD2 gene polymorphisms in tardive dyskinesia. Schizophr Res 106:248–52. https://doi.org/10.1016/j.schres.2008.08.036

Zai CC, Tiwari AK, Basile V, et al (2009a) Association study of tardive dyskinesia and five DRD4 polymorphisms in schizophrenia patients. Pharmacogenomics J 9:168–74. https://doi.org/10.1038/tpj.2009.2

Zai CC, Tiwari AK, Basile V, et al (2010a) Oxidative stress in tardive dyskinesia: genetic association study and meta-analysis of NADPH quinine oxidoreductase 1 (NQO1) and Superoxide dismutase 2 (SOD2, MnSOD) genes. Prog Neuropsychopharmacol Biol Psychiatry 34:50–6. https://doi.org/10.1016/j.pnpbp.2009.09.020

Zai CC, Tiwari AK, Chowdhury NI, et al (2019a) Genetic study of neuregulin 1 and receptor tyrosine-protein kinase erbB-4 in tardive dyskinesia. World J Biol Psychiatry 20:91–95. https://doi.org/10.1080/15622975.2017.1301681

Zai CC, Tiwari AK, De Luca V, et al (2009b) Genetic study of BDNF, DRD3, and their interaction in tardive dyskinesia. Eur Neuropsychopharmacol 19:317–28. https://doi.org/10.1016/j.euroneuro.2009.01.001

Zai CC, Tiwari AK, Mazzoco M, et al (2013) Association study of the vesicular monoamine transporter gene SLC18A2 with tardive dyskinesia. J Psychiatr Res 47:1760–1765. https://doi.org/10.1016/J.JPSYCHIRES.2013.07.025

Zai CC, Tiwari AK, Müller DJ, et al (2010b) The catechol-O-methyl-transferase gene in tardive dyskinesia. World J Biol Psychiatry 11:803–12. https://doi.org/10.3109/15622975.2010.486043

Zai CC, Tiwari AK, Zai GC, et al (2019b) Association Study of the Complement Component C4 Gene in Tardive Dyskinesia. Front Pharmacol 10:1339. https://doi.org/10.3389/fphar.2019.01339

Zhang XY, Zhang W-F, Zhou D-F, et al (2012) Brain-derived neurotrophic factor levels and its Val66Met gene polymorphism predict tardive dyskinesia treatment response to Ginkgo biloba. Biol Psychiatry 72:700–6. https://doi.org/10.1016/j.biopsych.2012.04.032

Zhang Z-J, Zhang X-B, Sha W-W, et al (2002a) Association of a polymorphism in the promoter region of the serotonin 5-HT2C receptor gene with tardive dyskinesia in patients with schizophrenia. Mol Psychiatry 7:670–1. https://doi.org/10.1038/sj.mp.4001052

Zhang Z, Zhang X, Hou G, et al (2002b) The increased activity of plasma manganese superoxide dismutase in tardive dyskinesia is unrelated to the Ala-9Val polymorphism. J Psychiatr Res 36:317–24. https://doi.org/10.1016/s0022-3956(02)00007-9
